# ER stress and Rho kinase activation underlie the vasculopathy of CADASIL

**DOI:** 10.1172/jci.insight.131344

**Published:** 2019-12-05

**Authors:** Karla B. Neves, Adam P. Harvey, Fiona Moreton, Augusto C. Montezano, Francisco J. Rios, Rhéure Alves-Lopes, Aurelie Nguyen Dinh Cat, Paul Rocchicciolli, Christian Delles, Anne Joutel, Keith Muir, Rhian M. Touyz

**Affiliations:** 1 Institute of Cardiovascular and Medical Sciences, University of Glasgow, United Kingdom.; 2 Institute of Neuroscience and Psychology, University of Glasgow and Queen Elizabeth University Hospital, Glasgow, United Kingdom.; 3 Golden Jubilee National Hospital, Clydebank, United Kingdom.; 4 Institute of Psychiatry and Neurosciences of Paris Inserm, Paris Descartes University, Paris, France.; 5 Kidney Research Centre, Ottawa Hospital Research Institute, University of Ottawa, Ottawa, Ontario, Canada.

**Keywords:** Vascular Biology, Signal transduction

## Abstract

Cerebral autosomal dominant arteriopathy with subcortical infarcts and leukoencephalopathy (CADASIL) leads to premature stroke and vascular dementia. Mechanism-specific therapies for this aggressive cerebral small vessel disease are lacking. CADASIL is caused by *NOTCH3* mutations that influence vascular smooth muscle cell (VSMC) function through unknown processes. We investigated molecular mechanisms underlying the vasculopathy in CADASIL focusing on endoplasmic reticulum (ER) stress and RhoA/Rho kinase (ROCK). Peripheral small arteries and VSMCs were isolated from gluteal biopsies of CADASIL patients and mesentery of TgNotch3^R169C^ mice (CADASIL model). CADASIL vessels exhibited impaired vasorelaxation, blunted vasoconstriction, and hypertrophic remodeling. Expression of *NOTCH3* and ER stress target genes was amplified and ER stress response, Rho kinase activity, superoxide production, and cytoskeleton-associated protein phosphorylation were increased in CADASIL, processes associated with Nox5 upregulation. Aberrant vascular responses and signaling in CADASIL were ameliorated by inhibitors of Notch3 (γ-secretase inhibitor), Nox5 (mellitin), ER stress (4-phenylbutyric acid), and ROCK (fasudil). Observations in human CADASIL were recapitulated in TgNotch3^R169C^ mice. These findings indicate that vascular dysfunction in CADASIL involves ER stress/ROCK interplay driven by Notch3-induced Nox5 activation and that *NOTCH3* mutation–associated vascular pathology, typical in cerebral vessels, also manifests peripherally. We define Notch3-Nox5/ER stress/ROCK signaling as a putative mechanism-specific target and suggest that peripheral artery responses may be an accessible biomarker in CADASIL.

## Abstract 2

ER stress and Rho kinase are potentially druggable targets in CADASIL, the most aggressive form of small vessel disease and dementia.

## Graphical abstract

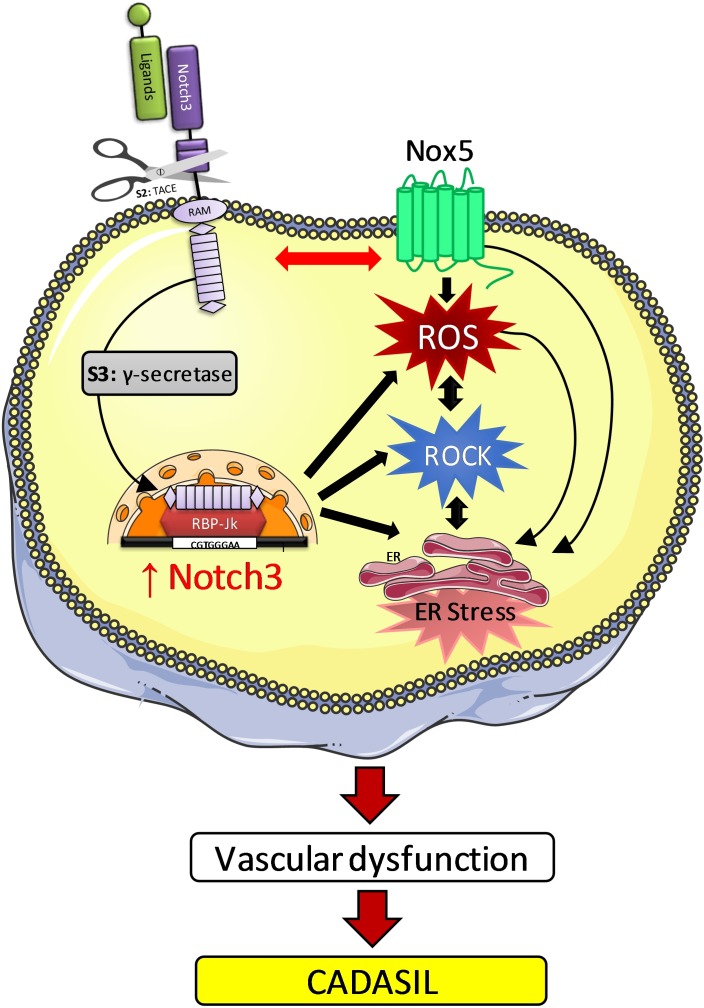

## Introduction

Cerebral autosomal dominant arteriopathy with subcortical infarcts and leukoencephalopathy (CADASIL) is the most aggressive form of small vessel disease of the brain leading to premature stroke and vascular dementia (1). It is caused by *NOTCH3* mutations (2) and is the commonest monogenetic form of stroke (3). CADASIL is a devastating condition because it affects adults in their prime years with the age of onset about 30 years, it is progressive, it is commonly associated with depression and psychiatric disorders, and there are no mechanism-specific treatments (1–4).

Although CADASIL manifests clinically as a vascular disease of the brain, all small- and medium-sized arteries are likely affected by CADASIL-causing *NOTCH3* mutations (5, 6). It is therefore possible that these mutations also cause vascular abnormalities peripherally. However, there is a paucity of information about the systemic microvasculature in CADASIL, with some studies reporting endothelial dysfunction in peripheral and retinal arteries, while others demonstrate normal endothelial function with altered vascular reactivity (7–12).

Notch3, composed of an extracellular domain (ECD), a transmembrane domain, and an intracellular domain (ICD), regulates the structural and functional integrity of small arteries. Unlike other Notch isoforms (Notch1, -2, and -4), Notch3 is expressed almost exclusively in vascular smooth muscle cells (VSMCs) (13). CADASIL-causing *NOTCH3* mutations lead to progressive degeneration of VSMCs, accumulation of abnormal protein (granular osmiophilic material [GOM]) around VSMCs, and vascular dysfunction (2, 14). In the brain, these processes present as subcortical lacunas and white matter injury and manifest clinically as premature stroke, dementia, cognitive decline, and migraines (1, 3, 15). While the clinical characteristics of CADASIL are well defined, the molecular and cellular processes underlying the vasculopathy are poorly understood.

Putative mechanisms have been studied in experimental models of CADASIL (TgNotch3^R169C^ mice) and immortalized human cell lines. Studies in TgNotch3^R169C^ mice demonstrated that cerebrovascular dysfunction is associated with upregulation of voltage-dependent potassium (Kv1) channels, blunted membrane depolarization, and reduced myogenic tone due to increased levels of metallopeptidase inhibitor TIMP3 (16–18). To date, these processes have not been demonstrated in human disease. Cell-based studies using cultured umbilical VSMCs from a CADASIL patient reported increased expression of proteins involved in protein degradation/folding, cytoskeletal organization, contraction, and cell stress (19). In human induced pluripotent stem cells generated from CADASIL somatic cells and in skin fibroblasts of CADASIL patients, increased platelet-derived growth factor (PDGF) signaling, enhanced TGF-β expression, endoplasmic reticulum (ER) retention of mutant Notch3 aggregates, and oxidative and ER stress have been described (20–24).

ER stress is a cellular response to the accumulation of unfolded/misfolded proteins in the ER and has been implicated in vascular dysfunction in cardiovascular disease (25, 26). Beyond its role in the processing of cellular proteins, ER stress influences VSMC function through its effects on Ca^2+^ handling, oxidative stress, and signaling molecules, including Rho kinase (27). Calcium and RhoA/Rho kinase are critically involved in controlling VSMC contraction, migration, growth, and cytoskeletal organization and increased Rho kinase activity has been demonstrated in many models of vascular dysfunction (28, 29).

A relationship between ER stress and Rho kinase has been demonstrated in VSMCs (30) and experimental models of vascular damage, where the ER stress response promotes activation of Rho kinase (30, 31). These phenomena may be regulated by Notch3-dependent processes because Notch3 associates with the protein-folding chaperone binding immunoglobulin protein (BiP; also known as GRP78) in the ER (32), mutations in Notch3 cause formation and retention of aggregates in the ER (23), and Notch3 modulates vascular RhoA/Rho kinase signaling (33). Accordingly, it is plausible that abnormal signaling through Notch3 and its downstream targets plays a role in vascular injury and dysfunction in CADASIL.

We hypothesized that aberrant Notch3 function promotes ER and oxidative stress and activation of RhoA/Rho kinase, which alter VSMC growth and cytoskeletal organization, processes leading to impaired vascular function in CADASIL. ER stress and Rho kinase are clinically important druggable targets because FDA-approved drugs that inhibit these targets are available. We interrogated these processes in disease-appropriate small arteries and VSMCs from clinically phenotyped CADASIL patients. We also used a reverse-translation approach in transgenic mice (TgNotch3^R169C^) that express the CADASIL-causing Notch3(R169C) mutant protein (6).

## Results

### Clinical features of subjects.

We studied 20 CADASIL patients (30–62 years old, 55% female), who carry between them 13 different *NOTCH3* genetic mutations, all of which involve cysteine residues encoded in exons 2 to 6. The clinical features, genetics, and demographic of the CADASIL patients are reported in Supplemental Table 2; supplemental material available online with this article; https://doi.org/10.1172/jci.insight.131344DS1 Eleven normotensive, healthy subjects on no medication and with no history of cardiovascular disease (34–58 years, 60% female) were studied as controls. Pulse wave velocity, which is an important clinical parameter to evaluate cardiovascular risk, was recorded in patients (7.84 ± 0.34 m/s) and found to be similar to published age-related normal values (34) and not different from our previously reported results in CADASIL patients (15). These findings indicate that the stiffness of large arterial vessels is not altered in CADASIL.

### Altered function, structure, and mechanical properties of peripheral small arteries in CADASIL.

**Figure 1:**
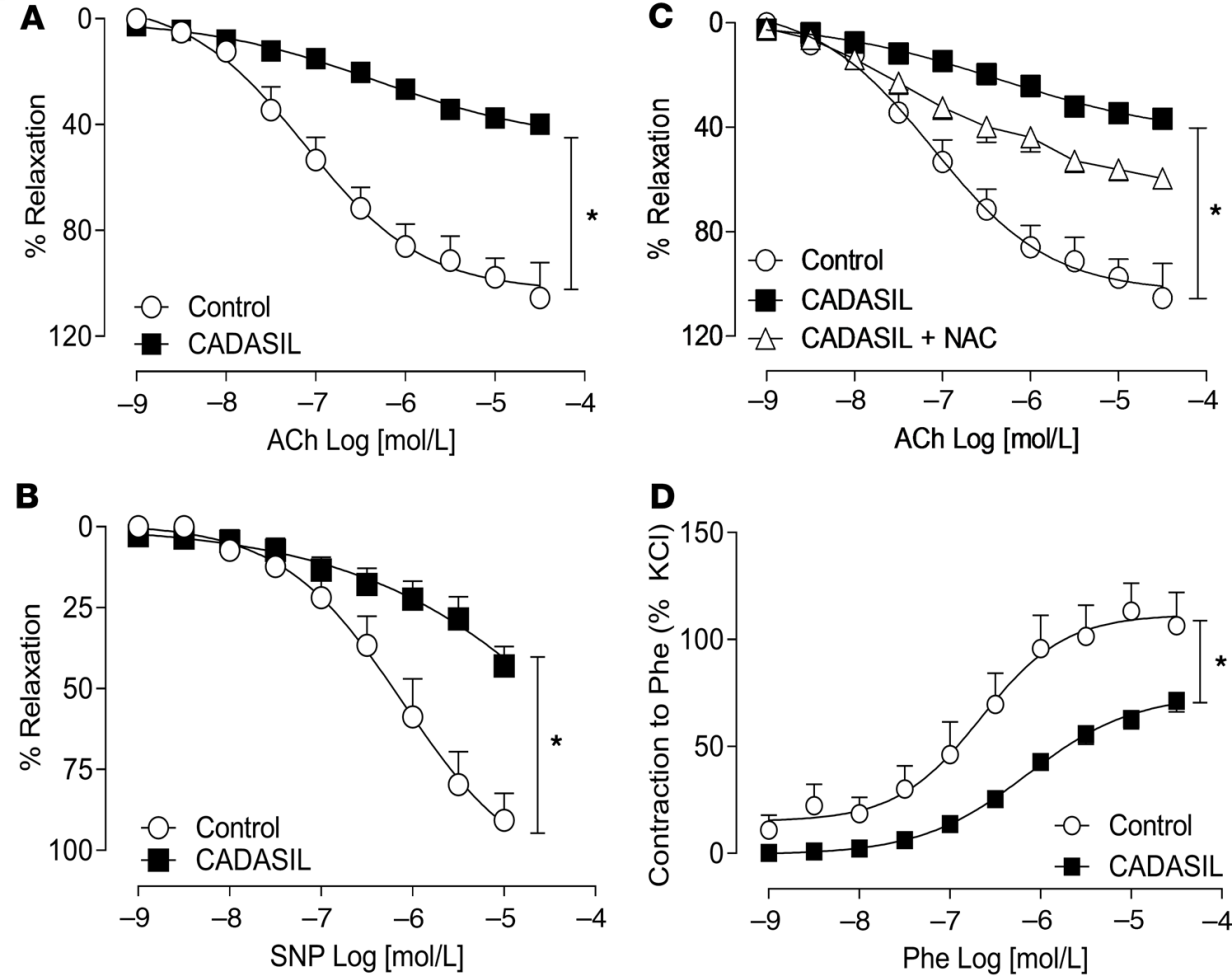
Reduced vasodilation and vasoreactivity in CADASIL arteries. Assessment of vascular functional responses in small arteries obtained from CADASIL and control subjects by wire myography. (**A**) Endothelium-dependent vasorelaxation in response to acetylcholine (ACh) and (**B**) endothelium-independent vasorelaxation in response to the nitric oxide (NO) donor, sodium nitroprusside (SNP), were decreased in CADASIL vessels (*n* = 6–17; two-way ANOVA with Bonferroni’s post hoc test). (**C**) The impairment in ACh-induced vasodilatation observed in CADASIL was slightly ameliorated in vessels pretreated with the ROS scavenger *N*-acetyl cysteine (NAC). (**D**) Maximum contractile responses (Emax) to phenylephrine (Phe) were also decreased in CADASIL vessels (*n* = 7–17; Student’s *t* test). Data are presented as mean ± SEM. *P < 0.05 versus control.

Small resistance arteries from gluteal biopsies were studied by wire and pressure myography to assess vascular contraction, relaxation, and structural properties. In vessels from healthy control subjects, maximal agonist-induced vasorelaxation (endothelium-dependent and -independent) and vasoconstriction were approximately 100% (Figure 1), as we and others have previously reported (35, 36). In small arteries from CADASIL patients, acetylcholine-induced (ACh-induced) vasorelaxation (endothelium-dependent) was significantly impaired, with maximal dilatory responses of 20%–30%, compared with 90%–100% in control vessels (Figure 1A). These effects in CADASIL were slightly ameliorated in vessels pretreated with the ROS scavenger *N*-acetyl cysteine (NAC) (Figure 1B). Sodium nitroprusside–induced (SNP-induced) vasorelaxation (endothelium-independent) (Figure 1C) was also significantly reduced relative to controls. Contractile responses to phenylephrine (PE) were decreased in CADASIL vessels compared with vessels from healthy controls (Figure 1D). Similar attenuated vasoconstriction was also evident for angiotensin II (Ang II), where maximal contractile responses were decreased in CADASIL vessels (Supplemental Figure 1).

**Figure 2:**
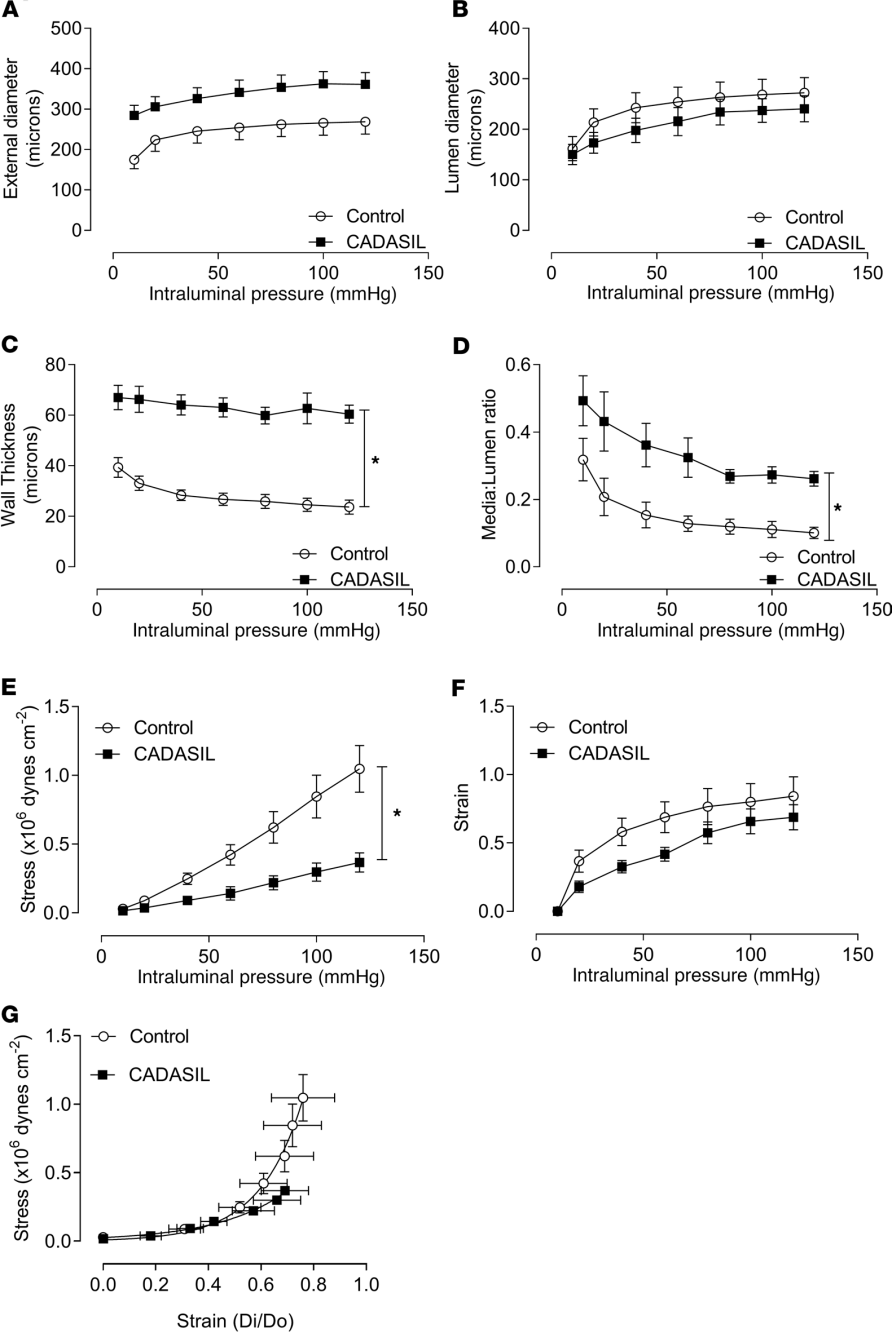
Vascular remodeling and altered mechanical properties in CADASIL arteries. Vascular structure and mechanical properties were assessed in pressurized small arteries obtained from CADASIL and control subjects. (**A**) External lumen diameter. (**B**) Lumen diameter. (**C**) Wall (media) thickness. (**D**) Media/lumen ratio. (**E**) Circumferential wall stress and (**F**) circumferential wall at increasing intraluminal pressure (10–120 mmHg) in calcium-free conditions. (**G**) Vascular stress-strain curve (*n* = 7–9; two-way ANOVA with Bonferroni’s post hoc test). Results are presented as mean ± SEM. *P < 0.05 versus control.

Vascular structure was altered in CADASIL subjects. While there were no changes in the external and internal diameters of CADASIL vessels relative to control vessels (Figure 2, A and B), the vascular wall (media) thickness and the media/lumen ratio were significantly greater in CADASIL vessels compared with controls (Figure 2, C and D), suggestive of hypertrophic remodeling (37). Mechanical properties (circumferential vessel wall stress, strain) were studied in pressurized vessels. Changes in circumferential vessel wall stress and strain under increasing intraluminal pressure showed a significant rightward shift in CADASIL versus control arteries, indicating reduced stiffness, or increased elasticity, in CADASIL vessels (Figure 2, E–G).

### VSMC phenotype is altered in CADASIL.

In order to further investigate the mechanisms underlying the vascular changes observed in CADASIL small arteries, primary culture VSMCs obtained from CADASIL vessels were studied. CADASIL VSMCs exhibited increased rates of cell growth, as measured by carboxyfluorescein succinimidyl ester (CSFE) and flow cytometry (Supplemental Figure 2, A and B) and by the increased expression of proliferating cell nuclear antigen (PCNA), a marker of cell proliferation (Supplemental Figure 2C). Caspase 3/7 activity, a molecular index of apoptosis, was also elevated in CADASIL VSMCs compared with controls (Supplemental Figure 1D). Phalloidin staining showed marked alteration of actin cytoskeletal fibers in CADASIL VSMCs (Supplemental Figure 3). Together, these data suggest a proliferative and proapoptotic profile with cytoskeleton disorganization in CADASIL VSMCs.

### Increased NOTCH3 signaling in CADASIL VSMCs.

**Figure 3:**
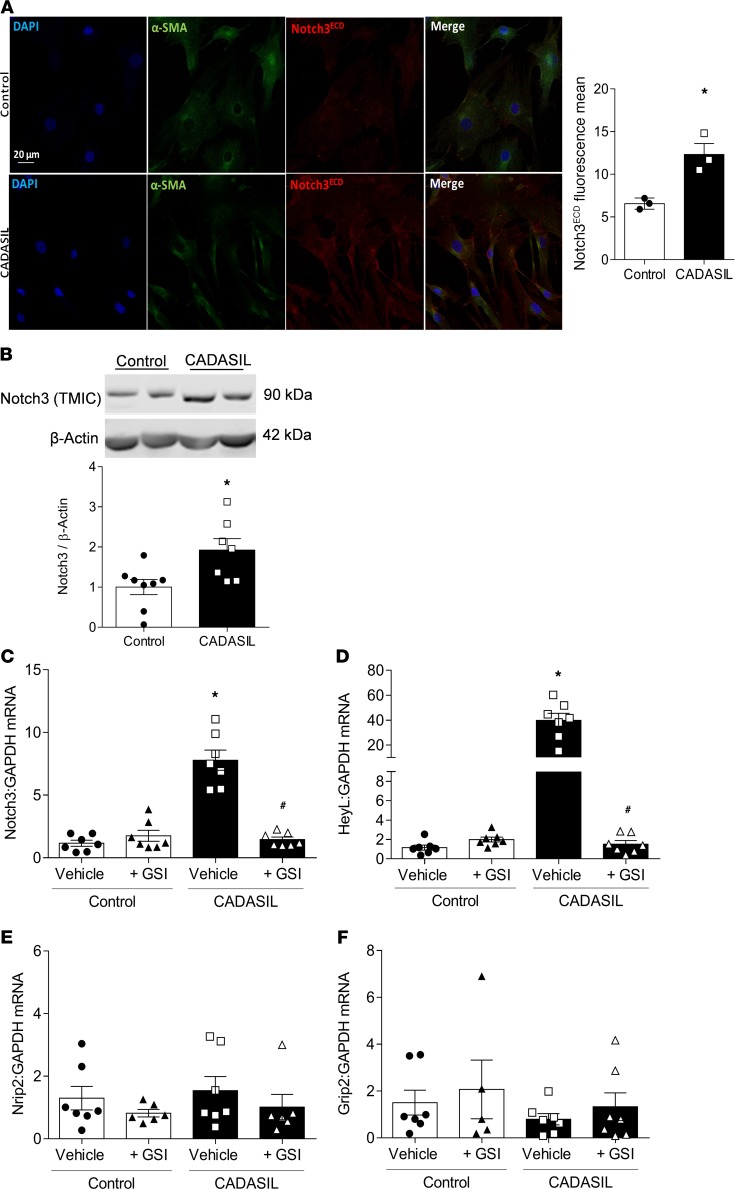
Mutant *NOTCH3* gain of function in CADASIL VSMCs. (**A**) Representative immunofluorescence image of Notch3^ECD^ in CADASIL and control VSMCs were stained with anti–α smooth muscle actin (α-SMA) and anti-Notch3^ECD^ antibodies. Objective: ×40. Scale bars: 20 μm. Corresponding bar graphs represent the fluorescence intensity of at least 5 images from 3 control and 3 CADASIL patients. (**B**) Expression of Notch3 transmembrane/cytosolic fragment (TMIC). Upper panel: Representative immunoblot. Lower panel: Quantification of Notch3 TMIC protein levels. Protein expression was normalized to β-actin (*n* = 7–8; Student’s *t* test). (**C**) *NOTCH3* gene expression and expression of NOTCH3 target genes (**D**) *HEYL*, (**E**) *Nrip2*, and (**F**) *Grip2* in CADASIL and control VSMCs in the absence and presence of a γ-secretase inhibitor (GSI). Analysis was by qPCR and gene expression was normalized to GAPDH (*n* = 5–8; two-way ANOVA with Bonferroni’s post hoc test). Results are expressed as mean ± SEM. *P < 0.05 versus vehicle control; ^#^*P* < 0.05 versus vehicle plus CADASIL.

To evaluate whether *NOTCH3* mutation is associated with impaired Notch3 function and signaling, we assessed Notch3 receptor and Notch3 ECD (Notch3^ECD^) levels as well as *NOTCH3*-downstream gene targets in VSMCs from control and CADASIL patients. CADASIL VSMCs exhibited increased Notch3^ECD^ accumulation (Figure 3A). Protein levels of Notch3 transmembrane/cytosolic fragment (TMIC) and *NOTCH3* receptor gene expression were increased in CADASIL VSMCs relative to controls (Figure 3, B and C). Following receptor cleavage, the Notch3 ICD (Notch3^ICD^) translocates to the nucleus, where it activates the transcription of Notch target genes, including hairy/enhancer-of-split related with YRPW motif–like protein (*HEYL*), nuclear receptor–interacting protein 2 (Nrip2), and glutamate receptor–interacting protein 2 (Grip2), which are involved in cell fate regulation. *HEYL* mRNA expression was increased in CADASIL VSMCs (Figure 3D). Expression of *NOTCH3* and *HEYL* mRNA in CADASIL VSMCs was decreased to control levels when cells were pretreated with a γ-secretase inhibitor (GSI), which inhibits Notch3 activation (Figure 3, C and D). No significant changes were observed for *Nrip2* and *Grip2* mRNA levels (Figure 3, E and F).

### Altered Ca^2+^ transients and augmented Rho kinase activity and ER stress in CADASIL.

**Figure 4:**
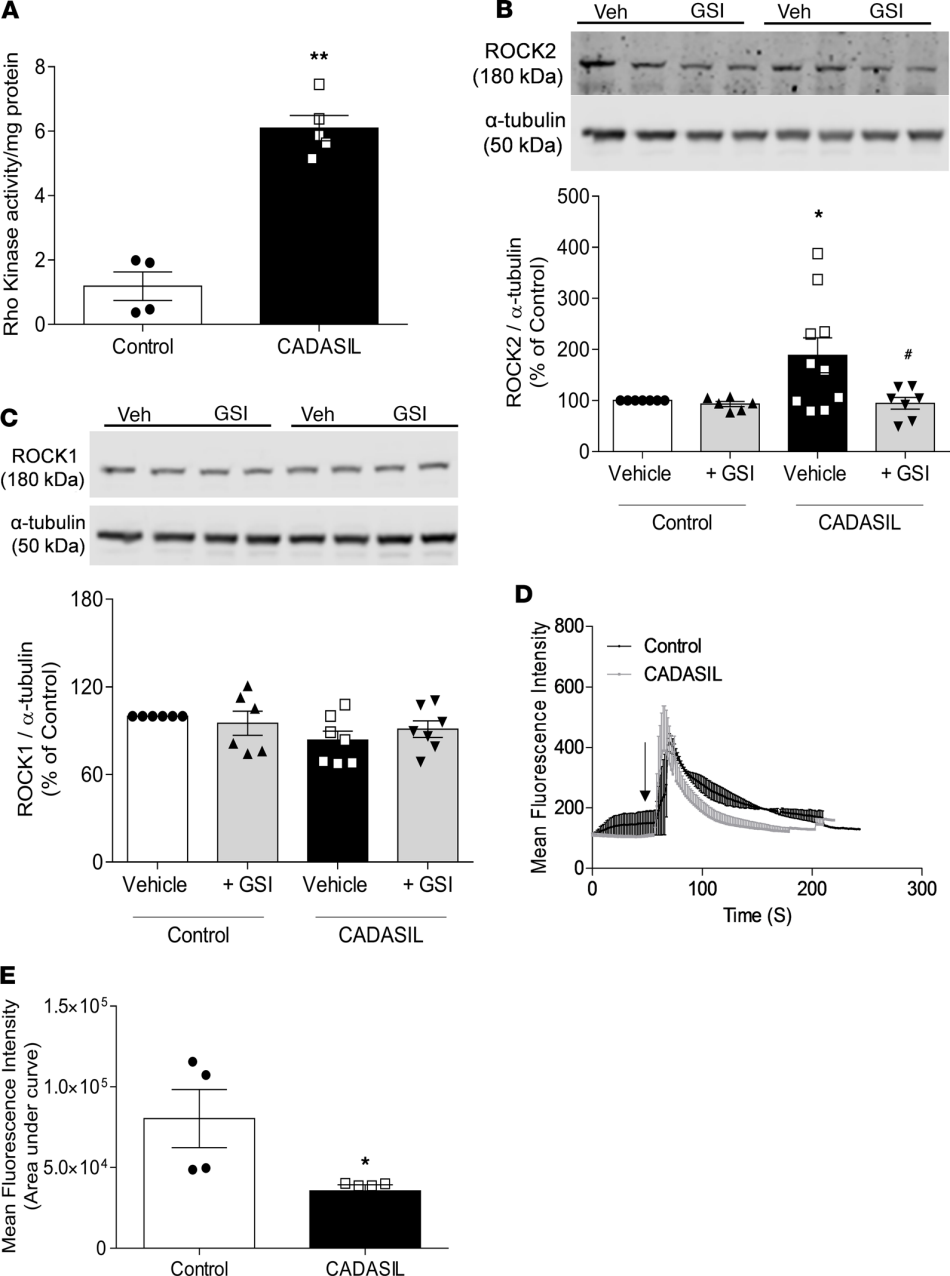
Increased RhoA/Rho kinase activity and blunted [Ca^2+^]_i_ transients in CADASIL VSMCs. (**A**) VSMC Rho kinase activity assessed by ELISA (*n* = 4–5; Student’s *t* test). (**B** and **C**) Expression of ROCK2 (**B**) and ROCK1 (**C**) in CADASIL and control VSMCs in the absence and presence of a γ-secretase inhibitor (GSI). Upper panels: Representative immunoblots. Lower panels: Quantification of ROCK2 and ROCK1 protein expression normalized to α-tubulin (*n* = 6–10; two-way ANOVA with Bonferroni’s post hoc test). (**D**) Calcium transients were measured by live cell fluorescence imaging using the fluoroprobe Cal-520 AM. Representative tracings of VSMC [Ca^2+^]_i_ responses to Ang II (1 × 10^–7^ mol/L) in CADASIL and control groups. Experiments were repeated 4 times/group with greater than 30 cells studied/field. (**E**) [Ca^2+^]_i_ calculated as the area under the curve. Arrow indicates time of Ang II stimulation (*n* = 4; Student’s *t* test). Results are expressed as mean ± SEM. *P < 0.05, **P < 0.01 versus control; ^#^*P* < 0.05 versus vehicle plus CADASIL.

To investigate possible mechanisms that underlie the vascular functional abnormalities observed in CADASIL vessels, we measured changes in agonist-stimulated Ca^2+^ responses and activation of the Rho kinase and ER stress pathways in VSMCs. As shown in Figure 4A, VSMC Rho kinase activity, as assessed by ELISA, was found to be increased in CADASIL VSMCs, relative to controls. This increased Rho kinase activity was associated with augmented protein expression of the Rho-associated kinase Rho kinase 2 (ROCK2), which was abolished when Notch signaling was inhibited by a GSI (Figure 4B). Protein expression of the other Rho-associated kinase, ROCK1, was unchanged (Figure 4C). Ca^2+^ transients were also altered in CADASIL patient–derived VSMCs, in which the agonist-induced Ca^2+^ response was acute and not sustained, compared with control VSMCs (Figure 4, D and E). This difference in the Ca^2+^ transient was reflected by reduced area under the curve in the CADASIL group, likely indicating decreased cytosolic [Ca^2+^]_i_.

**Figure 5:**
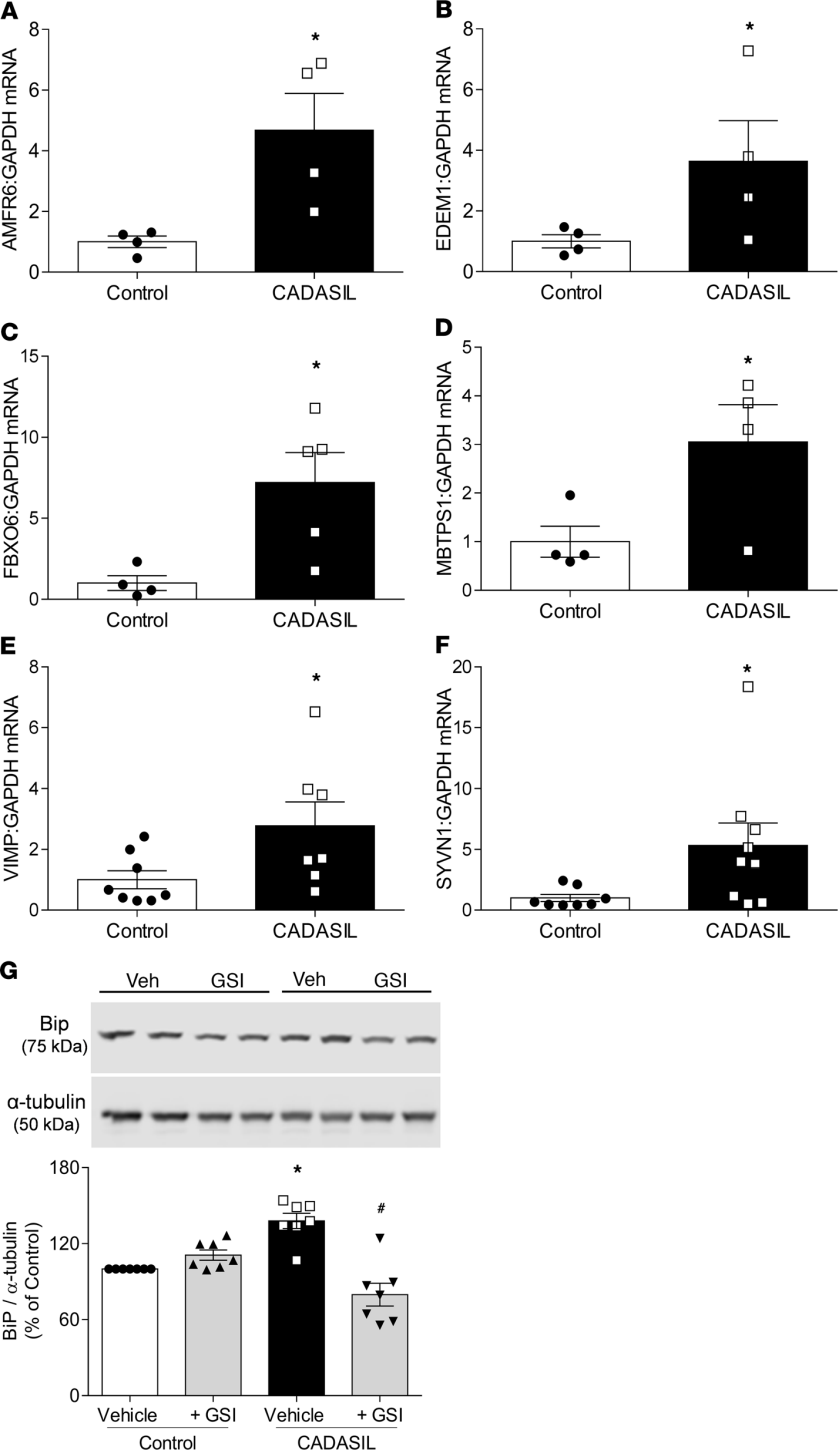
Increased ER and oxidative stress in CADASIL VSMCs. ER stress–related genes were assessed by qPCR: (**A**) *AMFR6*, (**B**) *EDEM1*, (**C**) *FBXO6*, (**D**) *MBTPS1*, (**E**) *VIMP*, and (**F**) *SYVN1*. Results are normalized to GAPDH and expressed as mean ± SEM (*n* = 4–9; Student’s *t* test). (**G**) Protein expression of binding immunoglobulin protein (BiP) (ER stress marker) in CADASIL and control VSMCs in the absence and presence of a γ-secretase inhibitor (GSI). Upper panel: Representative immunoblot of BiP. Lower panel: Quantification of BiP protein expression, normalized to α-tubulin (*n* = 7; two-way ANOVA with Bonferroni’s post hoc test). Results are expressed as mean ± SEM. *P < 0.05 versus vehicle control; ^#^*P* < 0.05 versus vehicle plus CADASIL.

**Figure 6:**
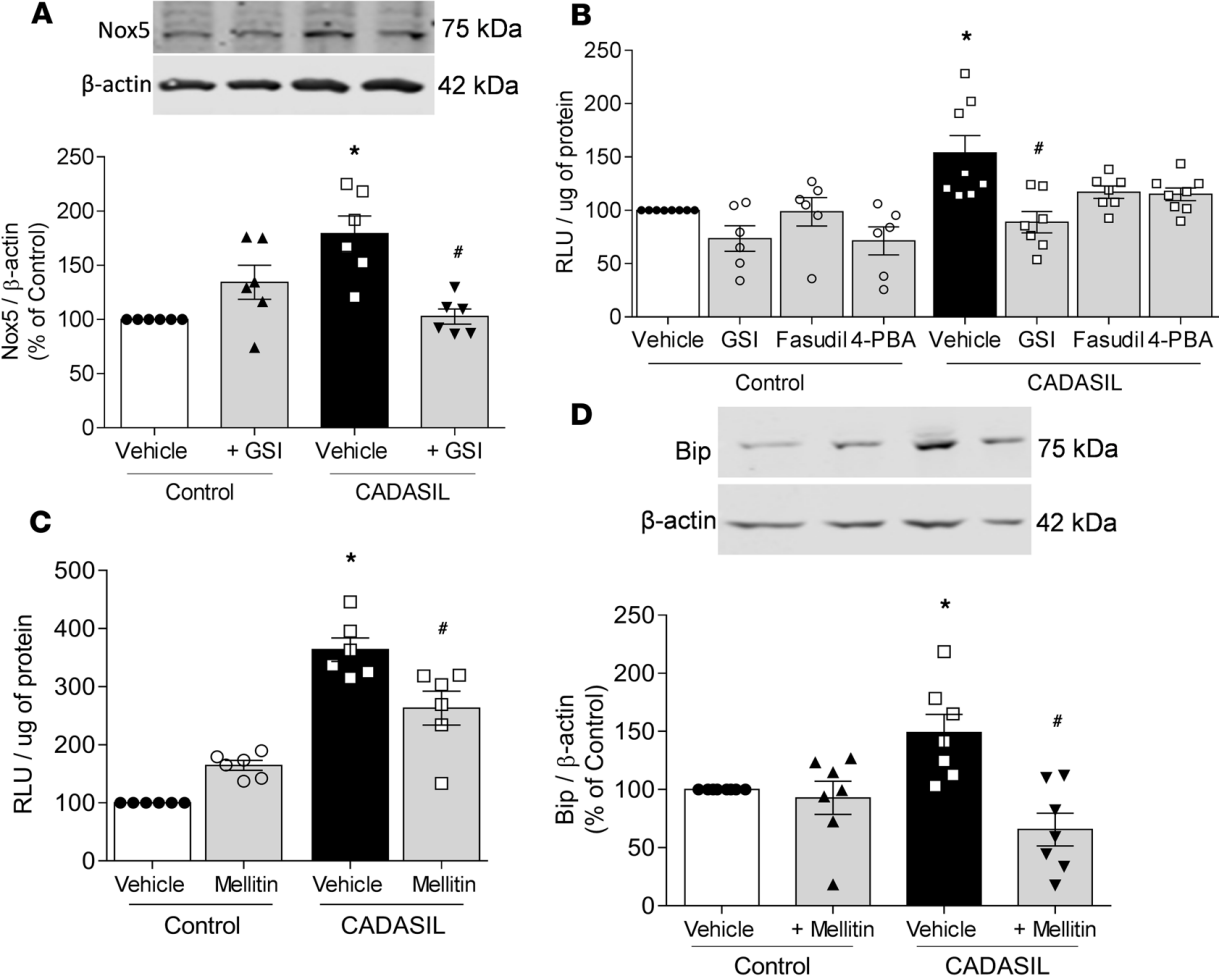
Nox5-derived ROS is an upstream regulator of Rho kinase and ER stress in CADASIL VSMCs. (**A**) Expression of Nox5 in CADASIL and control VSMCs in the absence and presence of a γ-secretase inhibitor (GSI). Upper panels: Representative immunoblots. Lower panels: Quantification of Nox5 protein expression normalized to β-actin (*n* = 6–10; two-way ANOVA with Bonferroni’s post hoc test). (**B**) ROS production measured by lucigenin in CADASIL and control VSMCs in the absence and presence of a GSI, fasudil, and 4-PBA. Results are normalized by protein content (*n* = 6–8; two-way ANOVA with Bonferroni’s post hoc test). (**C**) Lucigenin in CADASIL and control VSMCs in the absence and presence of a Nox5 inhibitor (mellitin). Results are normalized by protein content (*n* = 6; two-way ANOVA with Bonferroni’s post hoc test). (**D**) Expression of BiP in CADASIL and control VSMCs in the absence and presence of mellitin. Upper panels: Representative immunoblots. Lower panels: Quantification of BiP protein expression normalized to β-actin (*n* = 7; two-way ANOVA with Bonferroni’s post hoc test). Results are expressed as mean ± SEM. *P < 0.05 versus vehicle control; ^#^*P* < 0.05 versus vehicle plus CADASIL.

As shown in Figure 5, CADASIL patient VSMCs exhibited increased transcription of ER stress genes: autocrine motility factor receptor (*AMFR6*), ER degradation enhancer mannosidase α–like 1 (*EDEM1*), F-box protein 6 (*FBXO6*), membrane-bound transcription factor (*MBTPS1*), VCP-interacting membrane protein (*VIMP*), and synoviolin 1 (*SYVN1*) (Figure 5, A–F). CADASIL VSMCs also showed an increase in protein expression of BiP (Figure 5G), a molecular chaperone located in the ER and involved in ER protein handling. Increased BiP expression was reduced in CADASIL cells pretreated with the GSI. The ER stress response is typically associated with oxidative stress and, as shown in Figure 6, ROS production was significantly increased in CADASIL VSMCs compared with controls (Figure 6, B and C). Together, these data indicate that *NOTCH3* mutations in CADASIL VSMCs manifest as a gain of function and increased activation of Notch3, with associated upregulation of Rho kinase, ER stress responses, and oxidative stress.

### Nox5-derived ROS is an upstream regulator of Rho kinase and ER stress in CADASIL VSMCs.

Both Rho kinase activation and ER stress are redox-sensitive processes. Accordingly, we investigated whether ROS play a role in Rho kinase activation and the ER stress response in CADASIL VSMCs. As demonstrated in Figure 6A, expression of the major vascular Nox-producing oxidase in humans, NADPH oxidase 5 (Nox5), is increased in CADASIL. This was associated with significantly increased ROS generation (Figure 6, B and C), effects that were attenuated by GSI and mellitin (Figure 6, B and C), indicating that oxidative stress in CADASIL involves Notch3 and Nox5 and that Nox5 is downstream of Notch3. The increase in BiP expression observed in CADASIL VSMCs was also inhibited by mellitin (Figure 6D), suggesting that the ER stress observed in CADASIL is associated with Nox5.

### Interplay between NOTCH3, Rho kinase, and ER stress.

**Figure 7:**
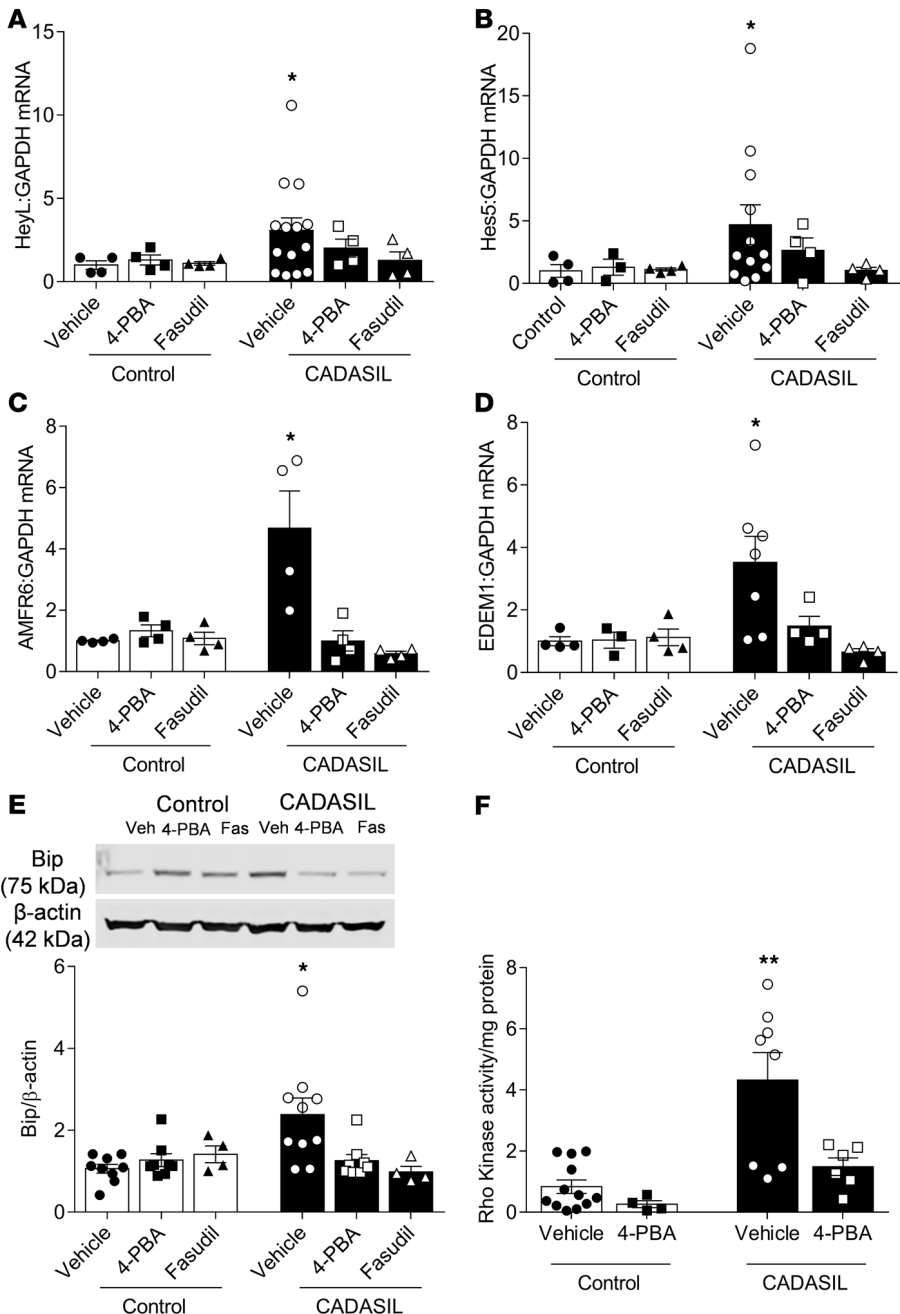
Interplay between Notch3, ER stress, and Rho kinase in CADASIL VSMCs. (**A**–**D**) Pretreatment of VSMCs with 4-PBA (1 × 10^–3^ mol/L) or fasudil (1 × 10^–5^ mol/L) reduced expression of (**A**) *HEYL* and (**B**) *HES5*, and ER stress–related genes (**C**) *AMFR6* and (**D**) *EDEM1* in CADASIL VSMCs without effect on control VSMCs (*n* = 3–14; one-way ANOVA with Bonferroni’s post hoc test). (**E**) Increased expression of BiP in CADASIL VMSCs was reduced in 4-PBA (1 × 10^–3^ mol/L) and fasudil (1 × 10^–5^ mol/L) pretreated cells. Upper panel: Representative immunoblot of BiP. BiP expression was normalized to β-actin (*n* = 4–10; one-way ANOVA with Bonferroni’s post hoc test). (**F**) 4-PBA decreased Rho kinase activity in CADASIL VMSCs, as assessed by ELISA (*n* = 4–12; one-way ANOVA with Bonferroni’s post hoc test). Results are expressed as mean ± SEM. *P < 0.05, **P < 0.005 versus vehicle group from control VSMCs.

To investigate potential interactions between Rho kinase and ER stress in CADASIL, we incubated VSMCs obtained from CADASIL patients and control subjects with 4-phenylbutyric acid (4-PBA, ER stress inhibitor) or with fasudil (Rho kinase inhibitor). Both 4-PBA and fasudil reduced expression of *NOTCH3* target genes *HEYL* (Figure 7A) and *HES5* (Figure 7B) in CADASIL VSMCs, an effect that was absent in control VSMCs. 4-PBA and fasudil also reduced expression of the ER stress markers, *AMFR6* (Figure 7C) and *EDEM1* (Figure 7D) in CADASIL, with no effect observed on control VSMCs. Treating CADASIL VSMCs with 4-PBA also significantly reduced levels of BiP (Figure 7E), and activity of Rho kinase (Figure 7F). 4-PBA did not significantly alter Ca^2+^ transients (Supplemental Figure 4).

### Rho kinase, ER stress, and VSMC function.

**Figure 8:**
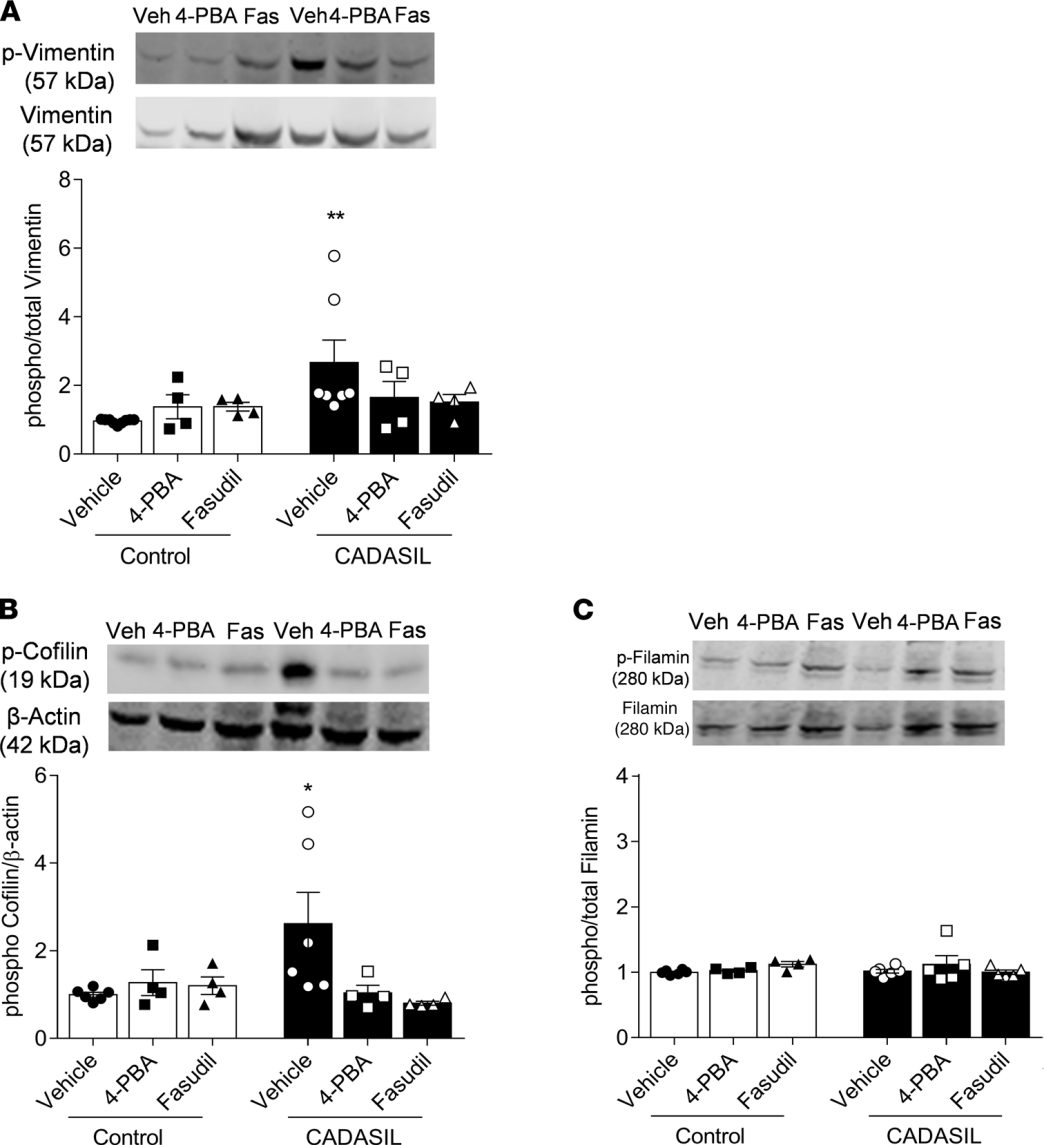
ER stress inhibition and Rho kinase activity ameliorate VSMC cytoskeletal regulation in CADASIL. (**A**–**C**) Phosphorylation of cytoskeleton-associated proteins vimentin (**A**), cofilin (**B**), and filamin (**C**) was assessed by Western blotting in CADASIL and control VSMCs in the absence and presence of 4-PBA (1 × 10^–3^ mol/L) and fasudil (1 × 10^–5^ mol/L). Representative Western blots show phosphorylated forms of vimentin, cofilin, and filamin. Expression levels of phosphorylated vimentin and filamin were normalized to total vimentin and filamin, while phosphorylated cofilin was normalized to β-actin. Results are expressed as mean ± SEM (*n* = 4–7; one-way ANOVA with Bonferroni’s post hoc test). *P < 0.05, **P < 0.005 versus control counterpart.

To determine whether the Notch3/Rho kinase/ER stress network contributes to the altered VSMC phenotype in CADASIL, we assessed effects of 4-PBA and fasudil on VSMC proliferation, apoptosis, and cytoskeletal regulation. Incubating CADASIL VSMCs with 4-PBA and fasudil did not influence their proliferation or apoptosis (Supplemental Figure 5, A and B) but ameliorated activation of cytoskeletal regulatory proteins. Specifically, increased phosphorylation of vimentin and cofilin in CADASIL VSMCs was normalized by 4-PBA and fasudil (Figure 8, A and B). In contrast, phosphorylation of filamin, another actin-binding protein, was not altered in CADASIL cells, and 4-PBA and fasudil had no effect on its expression or phosphorylation (Figure 8C). These data suggest that ER stress and Rho kinase play an important role in VSMC cytoskeletal regulation, without significantly influencing cell growth in CADASIL.

### Altered function of mesenteric arteries in TgNotch3^R169C^ mice.

TgNotch3^R169C^ mice were used as an experimental model to further dissect molecular pathways identified in CADASIL patients. As shown in Supplemental Table 3, body weight and systolic blood pressure (SBP) of TgNotch3^R169C^ mice were similar to wild-type (TgNotch3^WT^) controls. Cardiac function and structure were similar between groups, with no differences in heart weight, fractional shortening (FS), mitral valve E/A (ratio of early [E] and late [atrial; A] ventricular filling velocity), and anterior wall thickness between TgNotch3^R169C^ and control mice (Supplemental Table 3).

**Figure 9:**
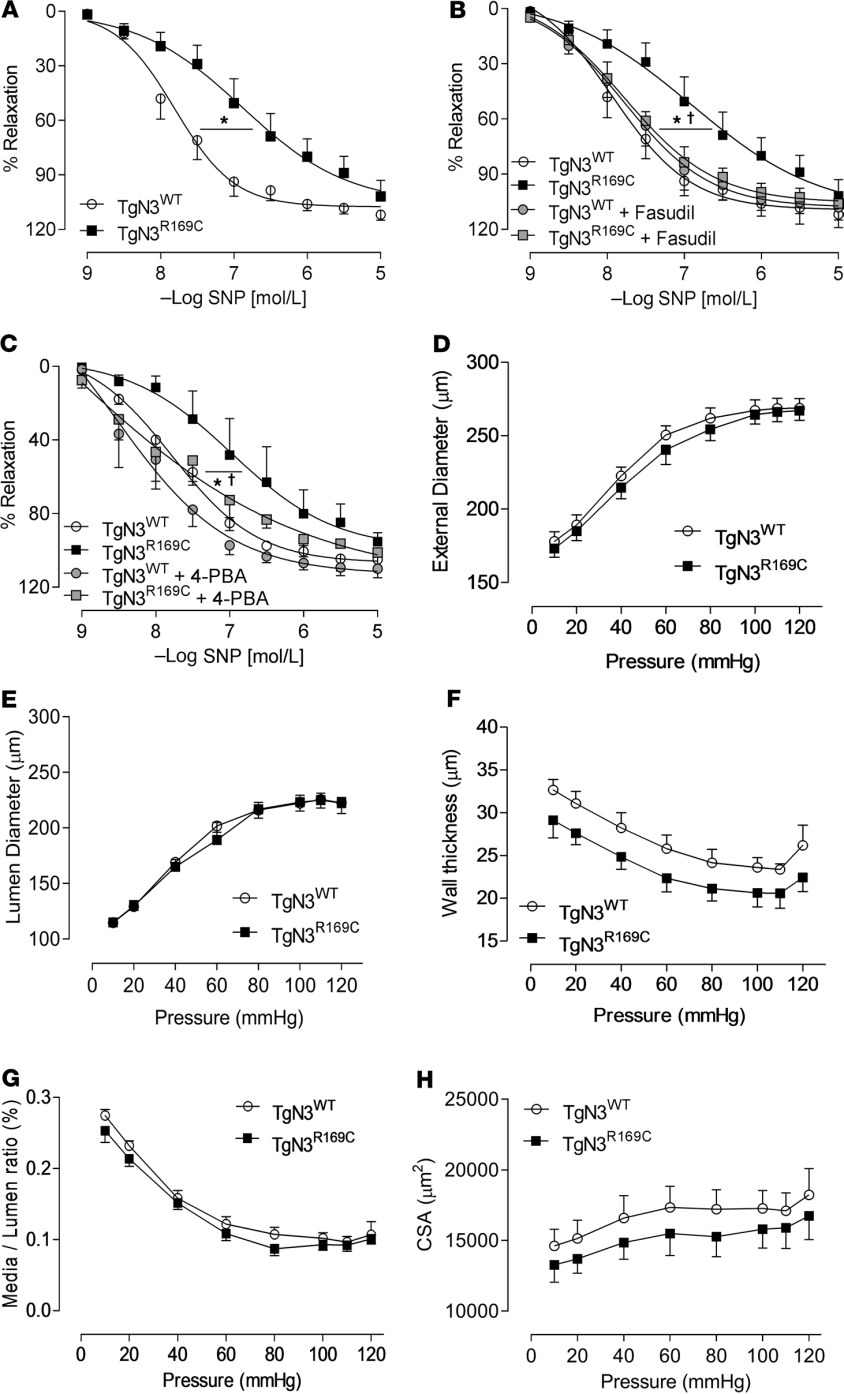
Vascular function and arterial remodeling in CADASIL mice. Assessment of vascular functional responses in mesenteric arteries from TgNotch3^WT^ (TgN3^WT^) and TgNotch3^R169C^ (TgN3^R169C^) mice by wire myography. (**A**) Endothelium-independent vasorelaxation in response to sodium nitroprusside (SNP). (**B**) Vasorelaxation in response to SNP in the presence of fasudil (1 × 10^–6^ mol/L; 30 minutes), and (**C**) 4-PBA (1 × 10^–3^ mol/L; 30 minutes) (2-way ANOVA with Bonferroni’s post hoc test). Vascular structure was assessed in pressurized mesenteric arteries from TgNotch3^WT^ and TgNotch3^R169C^ mice. (**D**) External lumen diameter. (**E**) Lumen diameter. (**F**) Wall thickness. (**G**) Media/lumen ratio. (**H**) Cross-sectional area (CSA) at increasing intraluminal pressure (10–120 mmHg) in calcium-free conditions. Results are presented as mean ± SEM (*n* = 6; one-way ANOVA with Bonferroni’s post hoc test). *P < 0.05 versus TgNotch3^WT^; ^†^*P* < 0.05 versus TgNotch3^R169C^.

We next assessed vascular function in small resistance arteries of TgNotch3^WT^ and TgNotch3^R169C^ mice using wire myography. Similar to what we observed in CADASIL patients, vessels from TgNotch3^R169C^ mice showed significant reduction in SNP-induced vasorelaxation (Figure 9A) compared with responses in TgNotch3^WT^ mice. These responses were restored when arteries were exposed to fasudil (Figure 9B) and 4-PBA (Figure 9C). No changes were observed in vessels from TgNotch3^WT^ mice incubated with fasudil and 4-PBA. We also investigated vascular structure by pressure myography. The external and internal diameters, cross-sectional area, wall thickness, and the media/lumen ratio of vessels in TgNotch3^R169C^ mice were similar to those in TgNotch3^WT^ vessels (Figure 9, D–H).

### Increased Notch3 signaling, Rho kinase and ER stress–related protein expression in TgNotch3^R169C^ mice.

**Figure 10:**
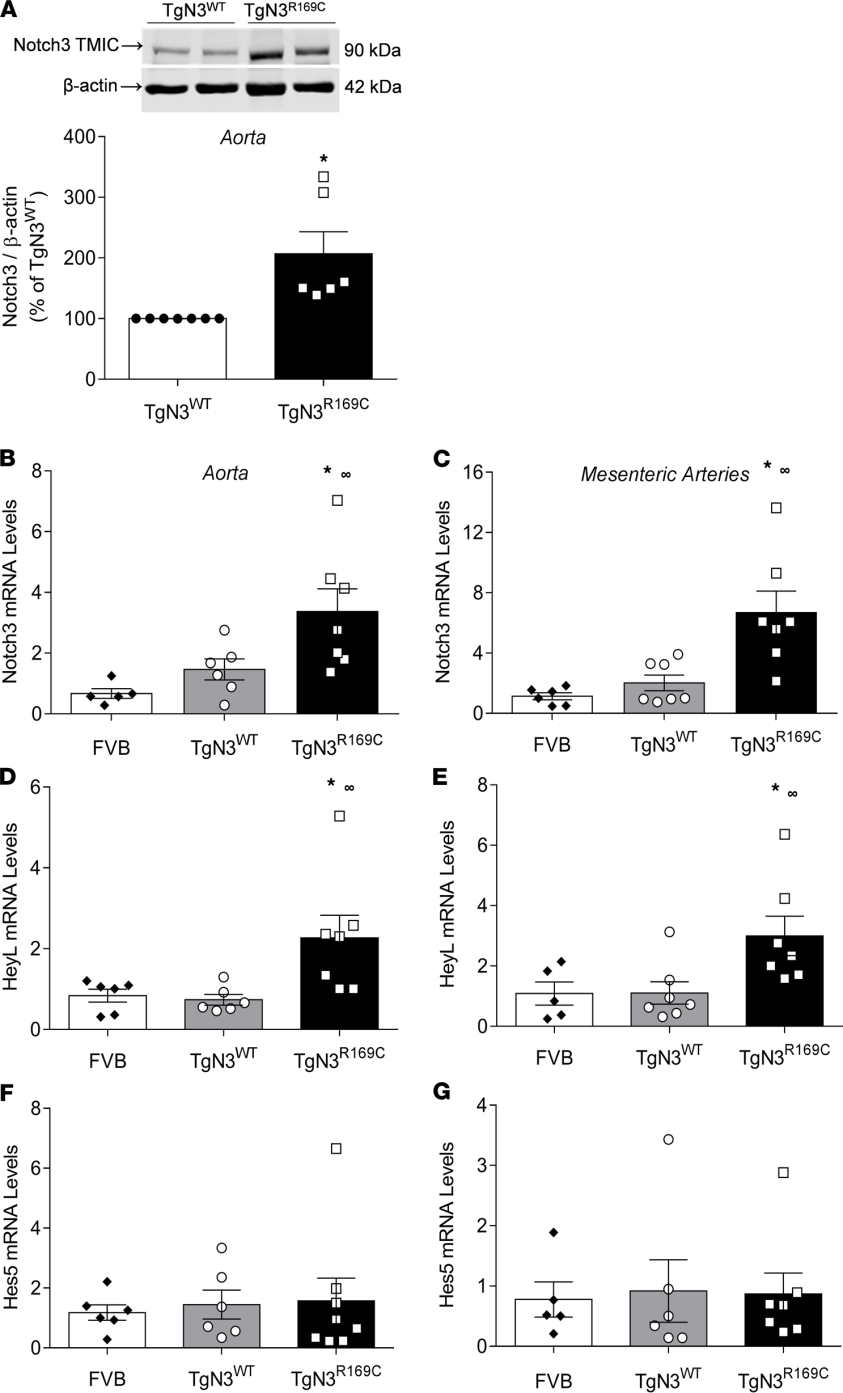
NOTCH3 signaling in mutant TgNotch3^R169C^ mice. (**A**) Upper panel: Representative immunoblot of the NOTCH3 transmembrane/cytosolic (TMIC) fragment in aortas from TgNotch3^WT^ (TgN3^WT^) and TgNotch3^R169C^ (TgN3^R169C^) mice. Lower panel: Quantification of NOTCH3 TMIC protein levels. Protein expression was normalized to β-actin (*n* = 6; Student’s *t* test). (**B**–**G**) *NOTCH3* gene expression and expression of NOTCH3 target genes *HEYL* and *Hes5* in FVB, TgNotch3^WT^, and TgNotch3^R169C^ aortas (**B**, **D**, and **F**) and mesenteric arteries (**C**, **E**, and **G**), as assessed by qPCR. Gene expression was normalized to GAPDH (*n* = 5–8; one-way ANOVA with Tukey’s post hoc test). Results are expressed as mean ± SEM. *P < 0.05 versus TgNotch3^WT^; ^∞^*P* < 0.05 versus FVB.

**Figure 11:**
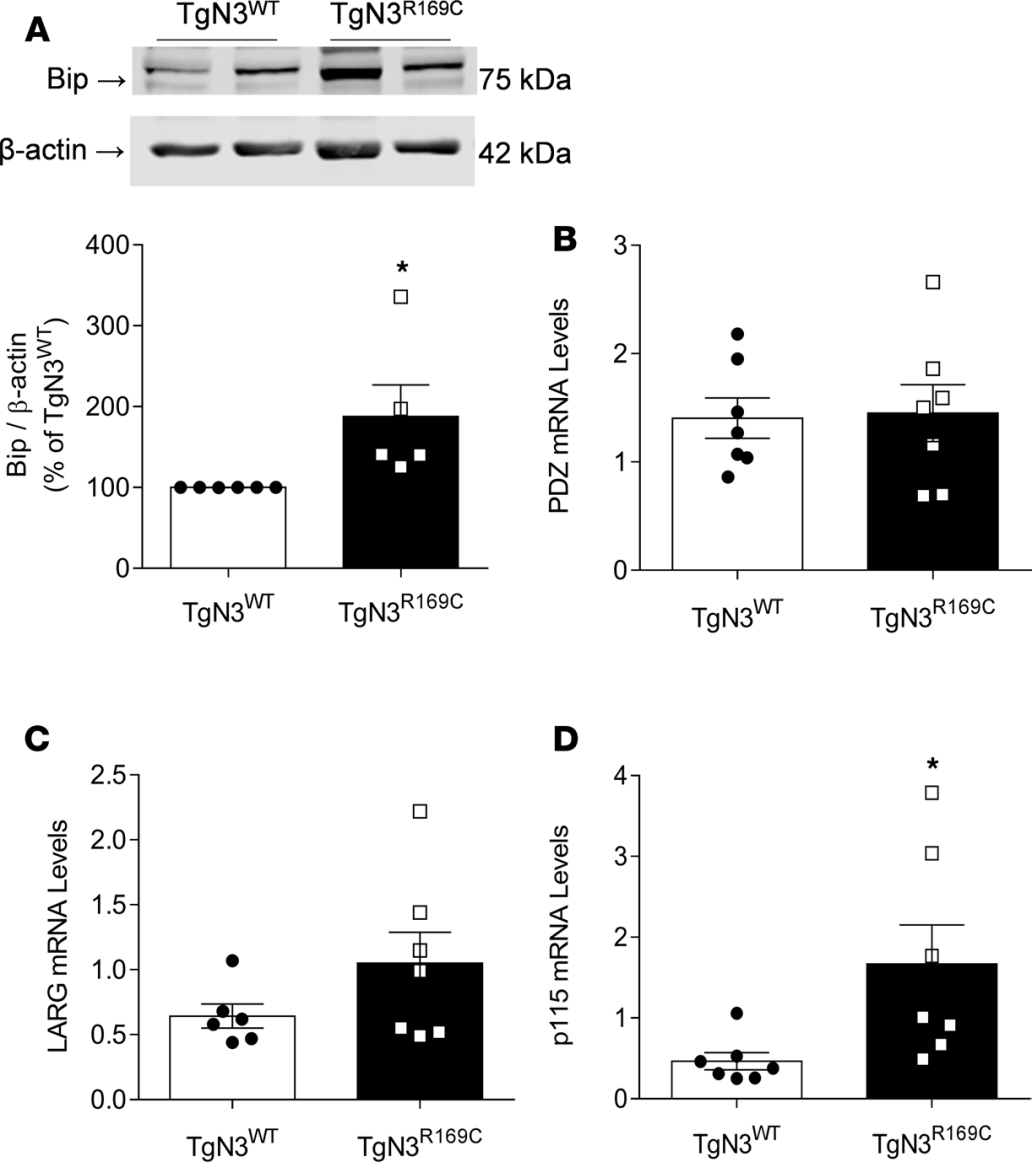
Increased vascular expression of ER stress– and Rho kinase–related proteins in CADASIL mice. (**A**) BiP protein levels were assessed by immunoblotting using aortas from TgNotch3^WT^ (TgN3^WT^) and TgNotch3^R169C^ (TgN3^R169C^) mice. Results are normalized to β-actin and are expressed as mean ± SEM (*n* = 5; Student’s *t* test). The same β-actin loading control was used in both **A** and Figure 10A since the same membrane was reprobed for β-actin, NOTCH3 TMIC, and BiP. (**B**–**D**) mRNA expression of the Rho GEFs PDZ (**B**), LARG (**C**), and p115 (**D**) in TgNotch3^WT^ and TgNotch3^R169C^ mice (*n* = 6–7; Student’s *t* test). Gene expression is normalized to GAPDH. Results are expressed as mean ± SEM. *P < 0.05 versus TgNotch3^WT^.

Expression of Notch3 TMIC was increased in TgNotch3^R169C^ vessels compared with TgNotch3^WT^ controls (Figure 10A). *Notch3* mRNA expression was also increased in the aorta and mesenteric arteries from TgNotch3^R169C^ mice compared with control wild-type mice (Figure 10, B and C, respectively). Transcription of the downstream *Notch3* target gene *Heyl* was increased in both the aorta (Figure 10D) and mesenteric arteries (Figure 10E) from TgNotch3^R169C^ mice compared with controls, while *Hes5* gene expression was unchanged in TgNotch3^R169C^ mice (Figure 10, F and G). As shown in Figure 11, A and D, respectively, vascular expression of BiP and the Rho guanine nucleotide–exchange factor (GEF), p115, was increased in TgNotch3^R169C^ mice compared with TgNotch3^WT^. Gene expression of *Larg* and *Pdz* was unchanged in TgNotch3^R169C^ mice (Figure 11, B and C).

## Discussion

Molecular processes underlying the vasculopathy in CADASIL are elusive. To address this, we investigated disease-appropriate human tissue from clinically phenotyped CADASIL patients and interrogated mechanisms at the molecular, cellular, and vascular levels. Major findings from our study show that small arteries in CADASIL exhibit functional and structural alterations defined by reduced vasoreactivity, hypertrophic remodeling, and decreased stiffness, processes linked to increased VSMC proliferation and apoptosis and cytoskeletal reorganization. At the molecular level, vascular abnormalities in CADASIL were associated with Notch3 gain of function, Nox5-induced oxidative stress, ER stress response, and Rho kinase activation. Perturbed signaling and activation of cytoskeleton-associated proteins in CADASIL were ameliorated by 4-PBA and fasudil, highlighting a role for ER stress and Rho kinase in the vasculopathy of CADASIL. These phenomena in human studies were recapitulated and further interrogated in TgNotch3^R169C^ mice, where vascular dysfunction was normalized by inhibitors of 4-PBA and fasudil. Our findings identify important interplay between Notch3, ER stress, and Rho kinase, in part through Nox5-derived ROS, that underlie small-vessel injury and dysfunction in CADASIL. These candidates may be potential mechanism-specific druggable targets in the disease.

We provide direct evidence that CADASIL-causing *NOTCH3* mutations have vascular manifestations beyond the cerebral vasculature and that peripheral small arteries are abnormal in CADASIL. Functionally, vasorelaxation was reduced and agonist-induced vasoconstriction was attenuated in CADASIL. VSMC contraction and relaxation are characteristically regulated by increased [Ca^2+^]_i_, which triggers contraction (38). Our findings of reduced agonist-stimulated Ca^2+^ transients indicate perturbed contractile signaling in CADASIL VSMCs. Mechanisms whereby mutant Notch3 influences these processes are unclear, but alterations in Ca^2+^ influx and efflux may be important since Notch3 regulates Ca^2+^ channels (store-operated Ca^2+^ entry, canonical transient receptor potential [TRPC6]) (39). Structurally, CADASIL arteries exhibited hypertrophic remodeling (increased media/lumen ratio) with reduced stiffness possibly due to cytoskeletal reorganization and dysregulated cell growth. Increased apoptosis was associated with VSMC growth in CADASIL and may represent attempts to counteract excessive proliferation and vascular remodeling. On the other hand, VSMC proliferation may be a response to cell death and apoptosis. Changes in the fine-tuning of cell growth and apoptosis probably contribute to structural vascular remodeling in CADASIL. Alteration in the VSMC phenotype in CADASIL may also be a direct effect of Notch3 activation because Notch3 plays an important role in phenotypic switch-type behavior of vascular cells, as demonstrated by a computational framework of Notch signaling (40). Confirming activation of Notch3 signaling in CADASIL cells was increased expression of NOTCH3 targets, HeyL and Hes, effects that were attenuated by γ-secretase inhibition.

Our findings of reduced vasoreactivity in peripheral arteries support our previous studies in cerebral arteries in CADASIL (15). Others have also reported decreased cerebrovasodilation in CADASIL patients challenged with CO_2_ and acetazolamide (41). These vascular abnormalities likely contribute to brain hypoperfusion injury and to the consequent stroke and dementia that characterize CADASIL. However, it remains unclear why CADASIL is not associated with systemic vascular disease (42), especially in light of the significant vascular dysfunction and arterial remodeling in the peripheral circulation, as we demonstrate here. It might be that systemic vasoprotective mechanisms, involving the sympathetic nervous system, differentially influence cerebral and peripheral arteries. It may also be possible that reduced vasoconstriction counteracts impaired vasodilation in an attempt to maintain vascular tone. This awaits confirmation, as yet undefined mechanisms are also likely to be important. CADASIL is typically a disease of small vessels, and in line with previous studies, vascular function of large arteries, as assessed by pulse wave velocity, was not altered in CADASIL patients in our study.

Experimental models of human disease, specifically TgNotch3^R169C^ mice that recapitulate salient properties of CADASIL, including cerebrovascular structural alterations, reduced vasodilation, and diminished pressure-induced vasoconstriction (6), have been used to dissect underlying mechanisms. Pathophysiological processes associated with cerebrovascular dysfunction in TgNotch3^R169C^ mice include a Kv1 channelopathy and increased levels of extracellular matrix proteins, including TIMP3 (6, 16–18, 43). These processes have not been demonstrated in human disease. Molecular systems that have been implicated in CADASIL patients include decreased TRPV1 channel expression, altered PDGF signaling, and oxidative stress (20–24). However, these studies were performed in human cell lines from the umbilical cord, skin fibroblasts, and induced pluripotent stem cells, which have little relevance in the vasculopathy of CADASIL (19–22).

In a proteomic screen in VSMCs from a CADASIL patient, expression of proteins involved in protein degradation/folding, cytoskeletal organization, contraction, and cell stress was increased (19). Many of these processes are associated with ER stress, which has also been demonstrated in CADASIL cells (23, 24) and in vascular dysfunction associated with hypertension (44) and diabetes (45). In our study, expression of ER stress genes, namely *AMFR6*, *EDEM1*, *FBXO6*, *MBTPS1*, *VIMP*, and *SYVN* was increased in CADASIL VSMCs. In addition, VSMC expression of BiP, a chaperone molecule involved in the ER unfolded protein response was increased in CADASIL patients, a phenotype that was recapitulated in TgNotch3^R169C^ mice. *AMFR6*, *EDEM1*, and *FBXO6* participate in protein degradation, while *MBTPS1*, *VIMP*, and *SYVN1* play a role in protein removal (46). Associated with ER stress in CADASIL VSMCs was upregulation of Rho kinase, which itself has been shown to influence ER stress (30). RhoA/Rho kinase signaling influences VSMC contraction, growth, and cytoskeletal organization and is an important regulator of Ca^2+^-independent vasocontraction and endothelium-independent vasorelaxation, processes that were impaired in CADASIL VSMCs. Increased Rho kinase activity has been demonstrated in models of vascular dysfunction (47, 48) and has been suggested as a therapeutic target in cerebral vascular disorders in patients with ischemic stroke and cerebrovascular disorders (49, 50). We advance this notion and suggest that Rho kinase plays a role in VSMC dysfunction in CADASIL. This is supported by studies showing that Notch3 is an upstream regulator of RhoA/Rho kinase and is involved in resistance artery mechanotransduction and pressure-induced (myogenic) tone (33, 51).

Pharmacological inhibition of Notch activation with GSI decreased expression of BiP and Rho kinase, confirming that Notch3 is an upstream regulator. The functional significance of these systems was further explored using 4-PBA and fasudil, which corrected many of the abnormal molecular changes in CADASIL VSMCs, including Notch3 downstream target genes, Rho kinase activity, and ER stress markers. In addition, in CADASIL VSMCs, 4-PBA and fasudil normalized increased phosphorylation of cytoskeleton-associated proteins, vinculin and cofilin, which are structural proteins that influence cytoskeletal organization. These inhibitors had no effect on VSMC proliferation or apoptosis but ameliorated the cytoskeletal remodeling defects in CADASIL, indicating that ER stress– and Rho kinase–mediated functional responses are not generalized phenomena, but are highly specific. Of significance, 4-PBA reduced Rho kinase activity, while fasudil decreased BiP expression, indicating crosstalk between ER stress and Rho kinase in the context of increased Notch3 activity in CADASIL. Interactions between these pathways have been reported in other systems, as evidenced by (i) reduction in renal Notch signaling in response to Rho kinase inhibition (52), (ii) Notch3-induced activation of Rho kinase in pluripotent stem cells (51), (iii) decreased vascular Rho A signaling in Notch3-deficient mice (33), (iv) amelioration of ER stress by Rho kinase inhibitors (30), and (v) Notch3 association with ER stress (32).

We further explored putative upstream systems regulating vascular signaling in CADASIL, focusing on oxidative stress, since Rho kinase and ER stress response are highly redox sensitive. Similar to what has been demonstrated previously in other small vessel diseases of the brain (53–56), we found increased ROS production in CADASIL VSMCs. Moreover, we identified Nox5, the major Nox isoform responsible for vascular ROS generation in humans, as a key source of vascular oxidative stress in CADASIL. Hence, we define what we believe is a novel system whereby mutant Notch3 activation induces Nox5-derived ROS generation that influences ER stress response and vascular function in human CADASIL. Mechanisms whereby Notch3 regulates Nox5 expression/activity are unclear but may relate to increased activation of Notch3-regulated transcription factors, which could influence Nox5 processing.

To further support the notion that Rho kinase and ER stress directly influence vascular dysfunction in CADASIL and as a proof of concept, we performed vascular functional studies in our preclinical mouse model of CADASIL and showed that both fasudil and 4-PBA ameliorate impaired vasorelaxation. The experimental model was studied to facilitate easy access of sufficient vascular tissue, which is a limitation in human studies. Whether similar effects observed in CADASIL mice also occur in intact vessels from CADASIL patients awaits confirmation, but our molecular studies in human CADASIL VSMCs are certainly supportive.

In conclusion, we demonstrate that peripheral small arteries from CADASIL patients exhibit endothelial dysfunction, impaired endothelium-independent vasorelaxation, hyporeactivity, and altered structural and mechanical properties, processes associated with altered VSMC growth and cytoskeletal reorganization and perturbed vascular signaling involving Notch3 gain of function, Nox5-derived ROS, ER stress, and Rho kinase activation. Using a reverse translational approach, observations in CADASIL patients were recapitulated in TgNotch3^R169C^ mice. Our findings identify a Notch3-regulated pathway involving interplay between the ER stress response and Rho kinase in the vasculopathy of CADASIL. Inhibiting ER stress and Rho kinase with FDA-approved pharmacological agents, 4-PBA and fasudil, ameliorated vascular dysfunction in CADASIL, highlighting these pathways as potentially beneficial therapeutic targets. Our study also shows that vascular alterations in CADASIL manifest peripherally and are not only confined to the cerebral vasculature, suggesting that peripheral artery responses may be an accessible biomarker in CADASIL.

## Methods

### Subject recruitment.

Patients with genetically and clinically confirmed CADASIL (*n* = 20) were recruited from the Neurovascular Genetics clinic, Queen Elizabeth University Hospital, Glasgow. Healthy controls were volunteers at the Ottawa Hospital Research Institute (*n* = 11). Under local anaesthetic, all subjects underwent a gluteal biopsy from which intact small arteries (<200 μm diameter) were dissected from subcutaneous fat. These arteries were used for functional, structural, and mechanical studies by wire and pressure myography, and VSMCs were isolated for primary cell culture, as previously described (35, 57) and summarized below. Identical protocols were used for CADASIL patients and control studies.

### Pulse wave analysis in CADASIL patients.

Pulse wave analysis was performed as an assessment of large vessel arterial stiffness using the Sphygmocor system, as previously described (15). Values were compared to published normal values for healthy control individuals (34).

### Mouse model of CADASIL.

The transgenic (Tg) mouse lines, TgNotch3^WT^ and TgNotch3^R169C^, have been previously characterized and described (6). Briefly, TgNotch3^WT^ and TgNotch3^R169C^ mice (on an FVB background) express rat wild-type Notch3 and the CADASIL-causing Notch3(R169C) mutant protein, respectively, to a similar degree (approximately 4-fold) compared with levels of endogenous Notch3 in nontransgenic mice (6). Mice were housed in individual cages in a room with controlled humidity and temperature (22°C–24°C), and in light/dark cycles of 12 hours with free access to food and tap water. They were studied at 6 months of age, at which stage features of CADASIL are well established, as previously described (6, 16, 17, 58, 59). Mice were sacrificed at 6 months of age (at which age they exhibit features of the human disease), and blood and tissue were collected for experiments. Small mesenteric arteries were used to assess vascular function, structure, and mechanics by wire and pressure myography. The aorta and mesenteric arteries were used to investigate Notch3 signaling and ER stress markers.

### Blood pressure measurement and echocardiography in mice.

SBP was assessed by tail-cuff plethysmography (60). Mice were trained to the apparatus (Visitech Systems model BP-2000) for 2 consecutive weeks until stable readings were obtained. SBP measurements were assessed on the week prior to the start of the experimental protocols.

Cardiac function and structure were assessed by echocardiography using an Acuson Sequoia c512 ultrasound system to acquire noninvasive 2D guided M-mode images at a 20 mm depth at the tip of the papillary muscles. Measurements were made in a short axis view using the leading edge-to-lead edge convention during both systole and diastole over at least 3 consecutive cardiac cycles. Echocardiographic indices assessed included left ventricular anterior wall thickness (LVAW), LV end-diastolic volume (LVEDV), LV end-systolic volume (LVESV), LV end-diastolic diameter (LVEDD), LV end-systolic diameter (LVESD), and FS to assess LV systolic function. Early (E) and late (atrial; A) ventricular filling velocity were calculated from measuring blood velocities across the mitral valve during each cardiac cycle. E/A ratio was calculated as an indirect measure of diastolic function. Ejection fraction (EF) = ([LVEDV – LVESV]/LVESD × 100); FS = ([LVEDD – LVESD]/LVEDD × 100).

### Human and mouse vascular functional studies.

Gluteal biopsies of subcutaneous fat measuring approximately 2 cm × 1 cm × 1 cm were obtained under local anaesthetic. Small arteries were dissected from gluteal fat and cut into 2-mm ring segments (34, 57). Small arteries were also dissected from mesenteric arteries isolated from TgNotch3^WT^ and TgNotch3^R169C^ mice, as previously described (57). Briefly, arterial segments were mounted on isometric wire myographs (Danish Myo Technology) filled with 5 mL of physiological saline solution (in mmol/L: 130 NaCl, 14.9 NaHCO_3_, 4.7 KCl, 1.18 KH_2_PO_4_, 1.17 MgSO_4_•7H_2_O, 5.5 glucose, 1.56 CaCl_2_•2H_2_O, and 0.026 EDTA) and continuously gassed with a mixture of 95% O_2_ and 5% CO_2_ while being maintained at a constant temperature of 37°C ± 0.5°C. Following 60 minutes of equilibration, the contractile responses of arterial segments were assessed by the addition of KCl (62.5 mmol/L). The integrity of the endothelium was verified by relaxation induced by Ach (1 × 10^–5^ mol/L) in arteries precontracted with PE (1 × 10^–6^ mol/L). Endothelium-dependent relaxation was assessed as a dose response to Ach (1 × 10^–9^ to 3 × 10^–5^ mol/L) in human vessels. Endothelium-independent vasorelaxation was assessed by a dose response to SNP (1 × 10^–9^ to 10^–5^ mol/L) in human vessels. Phenylephrine and Ang II (1 × 10^–9^ to 3 × 10^–5^ mol/L) concentration-response curves were generated to evaluate vasoconstriction in human arteries (60).

TgNotch3^WT^ and TgNotch3^R169C^ mouse vascular responses to SNP were performed in mesenteric arteries precontracted with U46619 (3 × 10^–8^ mol/L). Vascular functional responses to SNP were also assessed in the absence and presence of 4-PBA (1 × 10^–3^ mol/L, 30 minutes) or fasudil (1 × 10^–6^ mol/L, 30 minutes).

### Vascular structure and mechanical properties in human and mouse small arteries.

Additional arterial segments (~4 mm long) from CADASIL patients and control subjects and TgNotch3^WT^ and TgNotch3^R169C^ mice were studied as pressurized preparations using a Pressure Myograph System (110P system; Danish Myo Technology) in Ca^2+^-free physiological saline solution where intramural pressure was set to 70 mmHg for 30 minutes to allow vessels to equilibrate and to generate a variable degree of myogenic tone. Vessels were subjected to an increasing intramural pressure gradient, ranging from 10 to 120 mmHg at 5-minute intervals. Internal and external vessel wall measurements were recorded and used to calculate structural and mechanical properties, as previously described (26, 60). Briefly, from internal (D_i_) and external (D_e_) diameter measurements in passive conditions, the following structural parameters were calculated: wall thickness (WT) = (D_e_ – D_i_)/2; media/lumen ratio = (D_e_ – D_i_)/2D; circumferential wall strain = (D_i_ – D_o_)/D_o_, where D_o_ is the internal diameter at 10 mmHg and D_i_ is the observed internal diameter for a given intravascular pressure, both measured in Ca^2+^-free physiological saline solution; circumferential wall stress = (P × D_i_)/(2WT), where P is the intraluminal pressure (1 mmHg = 1.334 × 10^3^ dynes/cm^2^) and WT is measured at each intraluminal pressure in Ca^2+^-free physiological saline solution.

### Human VSMC isolation.

Methods for the isolation and culture of human VSMCs have been previously described (57). Briefly, cleaned arteries were placed in Ham’s F-12 culture medium containing 1% gentamicin, collagenase (type 1), elastase, soybean trypsin inhibitor, and BSA, and were incubated for 1 hour at 37°C under constant agitation. The digested tissue was further dissociated by repeated aspiration through a syringe with a 20-gauge needle. The cell suspension was centrifuged (2000 rpm, 4 minutes) and the cell pellet was resuspended in Ham’s F-12 culture medium containing 10% FBS. Cells were seeded onto 25-mm round glass coverslips. For the first 48 hours, cells were incubated in Ham’s F-12 culture medium containing 10% heat-inactivated fetal calf serum. Thereafter, VSMCs were maintained in Medium 231 containing Smooth Muscle Growth Supplement (SMGS) (both Thermo Fisher Scientific) with penicillin/streptomycin. Before experimentation, cells were rendered quiescent by maintenance in a reduced-growth-supplement medium (0.5% FBS) for 18 hours. Only primary, low-passage cells (passages 2 to 6) were studied. In some protocols, the role of ER stress, Rho kinase, and Notch signaling was assessed using pharmacological inhibitors: ER stress inhibitor, 4-PBA (1 × 10^–3^ mol/L, Sigma-Aldrich); Rho kinase inhibitor, fasudil (1 × 10^–5^ mol/L, Tocris); and Notch inhibitor, GSI XXIII (5 μmol/L, Calbiochem, 565792). Cells were preexposed to 4-PBA, fasudil, or GSI for 24 hours.

### Cell proliferation.

The fluorescein-based dye 5,6-carboxyfluorescein diacetate succinimidyl ester (CFSE) (CellTrace, Thermo Fisher Scientific) was used to assess proliferation of cultured VSMCs. CSFE is a fluorescent cell-staining dye used to monitor cell proliferation due to the progressive halving of CFSE fluorescence within daughter cells following each cell division. VSMCs were detached from culture plates using 0.04% Trypsin/0.03% EDTA (PromoCell GmbH, Germany) and were then incubated with 5 × 10^–6^ mol/L CFSE in 1 mL of PBS for 30 minutes at 37°C. Cells were then resuspended in complete Medium 231 and washed twice. CFSE-labeled VSMCs were plated at 30% confluence and cultured for 4 days in Medium 231, supplemented with 50% SMGS. After this period, cells were detached from the plates with 0.04% Trypsin/0.03% EDTA and resuspended in 1% BSA in PBS. Flow cytometry analysis with Ex/Em 492 nm/517 nm filters was performed using the FACS CANTO II system (BD Biosciences), using acquisition FACS DIVA software v6.1.3 (BD Biosciences). Data acquisition was analyzed using FlowJo X software (Tree Star).

### Apoptosis assay.

Apoptosis was assessed by caspase 3/7 activity measured using the Caspase-Glo 3/7 Assay kit (Promega) according to the manufacturer’s instructions. Briefly, 1000 VSMCs/well were seeded into Pierce White Opaque 96-Well Microplates (Thermo Fisher Scientific) and cultured for 3 hours to allow cells to adhere. The cells were then washed with PBS and the medium replaced with 50 μL Opti-MEM and incubated for 24 hours. An equal volume of the Caspase-Glo 3/7 substrate was added to each well of the cell culture plate and mixed using a microplate vortex mixer at maximum setting for 5 minutes to ensure cells were lysed. The plate was then incubated at room temperature for 1 hour before reading cell luminescence.

### Phalloidin staining.

Cytoskeletal organization was assessed using phalloidin staining (Cytoskeleton) according to the manufacturer’s instructions. Briefly, VSMCs were fixed with 2% paraformaldehyde (PFA) in PBS at room temperature for 20 minutes, PFA was removed, cells were washed 3 times with PBS, and nonspecific binding sites were blocked with 1% FBS in PBS for 30 minutes at room temperature followed by incubation with phalloidin stain (1 × 10^–7^ mol/L) in 1% FBS in PBS for 30 minutes in a humidified chamber in the dark. Cells were then washed 3 times with PBS and mounted on glass slides using Prolong medium with DAPI (Thermo Fisher Scientific). Fluorescence imaging was performed using a Zeiss confocal imaging system (LSM500). DAPI was excited at 405 nm and phalloidin at 535 nm. Images were acquired using Zen Pro (Zeiss). Each experimental group was imaged in duplicate with a minimum of 40 images being analyzed by 2 blinded observers using a previously described protocol (61) as follows: 1, “intact” filamentous structures in most cells (rare or no “damaged” fibrils); 2 (mild), more intact filamentous structure than damaged structures; 3 (moderate), more damaged structure than intact; and 4 (severe), predominantly damaged structures (few intact fibrils). Intact was defined as discernible, distinct, and organized filaments and damaged referred to disorganized, disarrayed, aggregated, clumped, or indiscernible structures.

### Quantitative real-time polymerase chain reaction.

Quantitative real-time polymerase chain reaction (qPCR) (Qiagen) was used to assess expression of Notch3 target genes (*Hes5* and *HeyL*) and ER stress genes (*AMFR6*, *EDEM1*, *FBXO6*, *MBTPS1*, *VIMP*, and *SYVN1*) in control and CADASIL VSMCs. mRNA expression of *Notch3*, *Hes5*, and *HeyL* was also measured by qPCR in the aorta and mesenteric arteries isolated from TgNotch3^WT^ and TgNotch3^R169C^ mice. The gene expression of the Rho GEFs LARG, p115, and PDZ was also analyzed in the aorta from TgNotch3^WT^ and TgNotch3^R169C^ mice. For some experiments, wild-type FVB mice were used as a control for Notch3 and Notch3 target gene expression because TgNotch3^WT^ and TgNotch3^R169C^ mice were on the FVB background. Briefly, total RNA was extracted from tissues using TRIzol (Qiagen), treated with RNase-free DNAse I, and 2 μg of RNA was reverse transcribed in a reaction containing 100 μg/mL oligo-dT, 10 mmol/L 2′-deoxynucleoside 5′-triphosphate, 5× First-Strand buffer, and 2 μL of 200-U reverse transcriptase. For real‑time PCR amplification, 3 μL of each reverse transcription product was diluted in a reaction buffer containing 5 μL of SYBR Green PCR master mix and 300 nmol/L primers in a final volume of 10 μL per sample. The reaction conditions consisted of 2 steps at 50°C for 2 minutes and 95°C for 2 minutes, followed by 40 cycles of 3 steps, 15-second denaturation at 95°C, 60-second annealing at 60°C, and 15 seconds at 72°C. Mouse primers used are detailed in Supplemental Table 1. Data are expressed as target gene/*GAPDH* housekeeping gene. Relative gene expression was calculated by the 2ΔΔCt method, and the results reported as arbitrary units relative to the control conditions. RT2 primer sets (Qiagen) were used (proprietary primers, sequence not disclosed). Human *GAPDH*, *Nrip2*, and *Grip2* primer sequences are as follows: Forward, GAGTCAACGGATTTGGTCGT; Reverse, TTGATTTTGGAGGGATCTCG; Forward, GTTCCTGCACAAGGACTCGG; Reverse, AGGCGTCTTTGGATCACACTG; and Forward, GCAGGGGAGACAATAGCGAAC; Reverse, CACAGTGATCCCTCGGAACT; respectively.

### Measurement of intracellular Ca^2+^ transients in VSMCs.

VSMC Ca^2+^ signaling was assessed using the fluorescent Ca^2+^ indicator, Cal-520 acetoxymethyl ester (Cal-520 AM; Abcam; 5 × 10^–6^ mol/L). Cells were grown in 6-well plates and following removal of culture media were incubated with 10^–5^ mol/L Cal-520 AM in 0.5% FBS at 37°C for 75 minutes followed by 30 minutes at room temperature. Following incubation, the dye solution was replaced with HEPES physiological saline solution (1.3 × 10^–1^ mol/L NaCl, 5 × 10^–3^ mol/L KCl, 10^–3^ mol/L CaCl_2_, 1 × 10^–3^ mol/L MgCl_2_, 2 × 10^–2^ mol/L HEPES, and 1 × 10^–2^ mol/L D-glucose, pH 7.4) for 30 minutes prior to imaging. VSMCs were stimulated with Ang II (1 × 10^–7^ mol/L) and the Ca^2+^ transient tracked for 5 minutes. In some experiments, VSMCs were pretreated for 24 hours with 4-PBA or fasudil. Fluorescence-based measurements of Ca^2+^ signals were performed using the inverted epifluorescence microscope Axio Observer Z1 Live-Cell imaging system (Zeiss) with excitation/emission wavelengths 490 nm and 525 nm, respectively. Images were acquired and analyzed using Zen Pro (Zeiss).

### Immunoblotting.

Protein was extracted from cultured human VSMCs and from vessels isolated from TgNotch3^WT^ and TgNotch3^R169C^ mice. Protein (30 μg) was separated by electrophoresis in a polyacrylamide gel and transferred to a nitrocellulose membrane. Nonspecific binding sites were blocked with 3% BSA in Tris-buffered saline (TBS) solution. Membranes were then incubated with specific antibodies overnight at 4°C. Membranes were washed 3 times with TBS/Tween 20 and incubated with infrared dye–labeled secondary antibodies for 1 hour at room temperature. Membranes were visualized using an Odyssey CLx infrared imaging system (LiCor Biosciences) and results were normalized to β-actin protein and are expressed in arbitrary units. Antibodies used were as follows: anti-ROCK2 (1:500, 610623, BD Biosciences); anti-ROCK1 (1:500; PIPA521130, Chemicon International); anti-PCNA (sc-56, Santa Cruz Biotechnology); anti-BiP (1:1000; 610978, BD Biosciences); anti-Notch3 (1:2000; 5276, Cell Signaling Technology); anti–β-actin (1:5000; A1978, Sigma-Aldrich); anti–α-tubulin (1:10000, ab4074, Abcam); anti–phospho-cofilin (3313S, Cell Signaling Technology); anti–phospho-vimentin (7391S, Cell Signaling Technology); anti-vimentin (3632S, Cell Signaling Technology); anti–phospho-filamin (4761S, Cell Signaling Technology); anti-filamin (4762S, Cell Signaling Technology); and anti-Nox5 (1: 1000; provided by David Harrison, Vanderbilt University Medical Center, Nashville, Tennessee, USA) (61).

### Rho kinase activity.

Rho kinase activity was measured with a Rho-associated Kinase Activity Assay kit (Merck, CSA001), according to the manufacturer’s instructions. Briefly, VSMCs cells were lysed and 50 μL was placed in 96-well multistrip plates and incubated for 30 minutes at 30°C on an orbital shaker. Each microwell was washed 3 times with 1× wash buffer before an anti–phospho-MYPT1 (Thr696) antibody was added for 1 hour at room temperature. Wells were washed again, and a goat anti–rabbit IgG HRP secondary antibody added for 1 hour at room temperature, followed by a wash and the addition of chromogenic substrate tetra-methylbenzidine (TMB) for 15 minutes. The reaction was stopped with Stop Solution and absorbance at 450 nm was measured spectrophotometrically. Rho kinase activity was normalized to protein content for each sample measured using the DC assay (Bio-Rad).

### Immunofluorescence staining of VSMCs.

For immunofluorescence analysis, VSMCs were plated and grown on a microscope chamber slide (Ibidi). Cells were fixed in fresh 2% PFA for 20 minutes followed by washing with PBS. Nonspecific binding was blocked with 5% BSA in PBS for 1 hour at room temperature. Primary antibody incubation was performed overnight at 4°C with the following antibodies diluted in 0.2% BSA in PBS: mouse monoclonal anti-Notch3^ECD^ (clone 1E4, 1:200, MABC594, Millipore) and rabbit polyclonal anti–α smooth muscle actin (1:200, ab5694, Abcam). For detection, Alexa Flour 488– and Alexa Flour 647–coupled secondary antibodies (Life Technologies) were diluted in 0.2% BSA in PBS and applied for 1 hour at room temperature. A mouse IgG1 negative control antibody (Serotec, MCA928) was also used. After washing, vessels were mounted with Fluoroshield mounting medium containing DAPI (Abcam, ab104139). Images were captured by confocal microscopy (LSM880, Zeiss) using a 40× objective. The fluorescence mean value was determined using ImageJ (version 1.60, NIH).

### Lucigenin-enhanced chemiluminescence.

Lucigenin-enhanced chemiluminescence assay was used to determine NADPH-dependent ROS production in control and CADASIL VSMCs, as we previously described (62). Cells were homogenized in lysis buffer (20 mmol/L KH_2_PO_4_, 1 mmol/L EGTA, 1 μg/mL aprotinin, 1 μg/mL leupeptin, 1 μg/mL pepstatin, and 1 mmol/L PMSF). Fifty microliters of the sample was added to a suspension containing 175 μL of assay buffer (50 mmol/L KH_2_PO_4_, 1 mmol/L EGTA, and 150 mmol/L sucrose) and lucigenin (5 μmol/L). Luminescence was measured with a luminometer (AutoLumat LB 953, Berthold) before and after stimulation with NADPH (100 μmol/L). A buffer blank was subtracted from each reading. The results are expressed in arbitrary units per milligram of protein, as measured by the BCA assay.

### Statistical analysis.

Data are expressed as mean ± SEM unless otherwise stated. Statistical comparisons of parameters between groups were performed using 2-tailed Student’s *t* test, or 1-way and 2-way ANOVA followed by Bonferroni’s post hoc tests as appropriate. *P* < 0.05 was considered statistically significant. Repeated-measures ANOVA was used for comparison of groups within vascular reactivity studies. Data analysis was conducted using GraphPad Prism 5.0.

### Study approval.

Ethics approval for the use of human samples was obtained from the West of Scotland Research Ethics Service (WS/12/0294) and from the Ethics Board of the Ottawa Hospital Research Institute, Canada (no. 997392132). Written informed consent was obtained for all study participants in accordance with the Declaration of Helsinki. All experimental protocols using mice were performed in accordance with the Ethical Principles in Animal Experimentation adopted by the West of Scotland Research Ethics Service and in accordance with the United Kingdom Animals Scientific Procedures Act 1986 and ARRIVE guidelines and approved by the institutional ethics review committee (70/9021).

## Author contributions

APH, KBN, ACM, FJR, and RMT designed the experiments. APH, RAL, KBN, FM, PR, ANDC, and FJR conducted experiments and acquired and analyzed data. AJ, CD, and RMT provided critical analyses of the manuscript. FM, CD, and KM provided clinical information and access to patients. APH, KBN, and RMT wrote the manuscript. The study was conceived and funding managed by RMT.

## Supplementary Material

Supplemental data

## Version 1 10/24/2019

In-Press Preview

## Version 2 12/05/2019

Electronic publication
