# Supplementary material for: ER stress and Rho kinase activation underlie the vasculopathy of CADASIL
Source: JCI Insight. 2019 Dec 5;4(23):e131344. doi: 10.1172/jci.insight.131344 (PMC6962020; doi:10.1172/jci.insight.131344)
Supplement: Supplemental data [file jciinsight-4-131344-s094.pdf]

SUPPLEMENTAL TEXT *MS#:131344-INS-RG-RV-3*

ER stress and Rho kinase activation underlie the vasculopathy of CADASIL

*Karla B Neves[1], *Adam P Harvey[1], Fiona Moreton[2], Augusto C Montezano[1], Francisco J Rios[1], Rhéure Alves-Lopes[1], Aurelie Nguyen Dinh Cat[1], Paul Rocchicciolli[3], Christian Delles[1], Anne Joutel[4], Keith Muir[2], Rhian M Touyz[1,5]. *Co-first authors

[1]Institute of Cardiovascular and Medical Sciences, University of Glasgow, UK;

[2]Institute of Neuroscience & Psychology, University of Glasgow & Queen Elizabeth University Hospital; [3] Golden Jubilee National Hospital, Clydebank, UK;

[4] Institute of Psychiatry and Neurosciences of Paris, Inserm, Paris Descartes University, France; [5]Kidney Research Centre, Ottawa Hospital Research Institute, University of Ottawa, Canada.

Conflicts of interest: There are no conflicts of interest to declare.

To whom correspondence should be addressed:

Rhian M Touyz MD, PhD

Institute of Cardiovascular and Medical Sciences,

University of Glasgow,

126 University Place, Glasgow, G12 8TA,

Glasgow, UK

Phone: + 44 (0)141 330 7775/7774, Fax: + 44 (0)141 330 3360,

Email: Rhian.Touyz@glasgow.ac.uk

Supplementary table 1. List of mouse primers

Gene	Sense primer	Anti-sense primer
Gapdh	AGGTCGGTGTGAACGGATTTG	TGTAGACCATGTAGTTGAGGTCA
Notch3	TGCCAGAGTTCAGTGGTGG	CACAGGCAAATCGGCCATC
HeyL	CAGCCCTTCGCAGATGCAA	CCAATCGTCGCAATTCAGAAAG
Hes5	AGTCCCAAGGAGAAAAACCGA	GCTGTGTTTCAGGTAGCTGAC
Larg	CGTTGGTCTGGAAGGTGAAT	CACCGTGCTCAGCTTAATGA
p115	TCCGGACCAAGAGTGGGGACAAGA	TCTCATCAGCCTCGACCTTT
Pdz	GAGTCTCGACCTTCCAGCAC	CTCTTGGGCTTCCCAATGTA

Supplementary table 2. Clinical characteristics of patients with CADASIL

**Characteristics**	**Cohort**	
Demographic characteristics		
Age, median (range), years	52	(30-62)
Female, n (%)	11	(55)
Measurements		
Systolic blood pressure mmHg, median (IQR)	129	(21)
BMI kg/m^2, median (IQR)	28	(5.6)
eGFR >60, n (%)	20	(100)
Clinical Features, n (%)		
Stroke or TIA	9 /10	(45-50)
Migraine	15	(75)
Depression	9	(45)
Vascular Risk Factors, n (%)		
Current or ex-smoker	10	(50)
Hypertension	1	(5)
Hypercholesterolaemia	3	(15)
Diabetes mellitus	1	(5)
Medication, n (%)		
Statin	16	(73)
Antiplatelet	18	(90)
Beta-blocker[$]	1	(5)
Diuretic	3	(15)
ACE-inhibitor	2	(10)

Supplementary table 3.

Characteristics of TgNotch3^{WT} and TgNotch3^{R169C} mice

**Parameter**	**TgN3^{WT} (n=6)**	**TgN3^{R169C} (n=6)**
Body Weight (g)	30.4 ± 0.7	30.5 ± 1.3
Systolic Blood Pressure (mmHg)	123.2 ± 4.6	121.5 ± 4.3
Heart Weight / Tibia Length (mg/cm)	110.1 ± 6.6	97.8 ± 5.0
Fractional Shortening (%)	41.5 ± 4.9	43.4 ± 4.6
Mitral Valve E/A	1.7 ± 0.3	1.37 ± 0.1
Anterior Wall Thickness	1.4 ± 0.1	1.6 ± 0.1

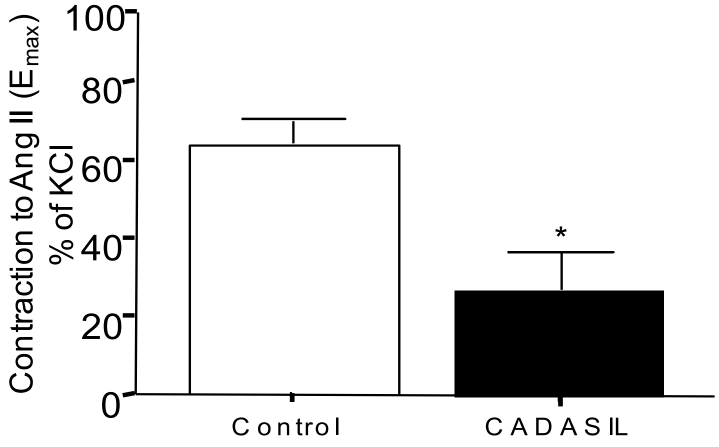

Figure S1. Reduced vasoreactivity in CADASIL arteries. Vascular functional responses to angiotensin II (Ang II) in small arteries from CADASIL and control subjects were assessed by wire myography. Bar graphs demonstrate the maximum contractile response, Emax, to Ang II. Data are presented mean±SEM. (n=6/group). *p<0.05 vs Control.

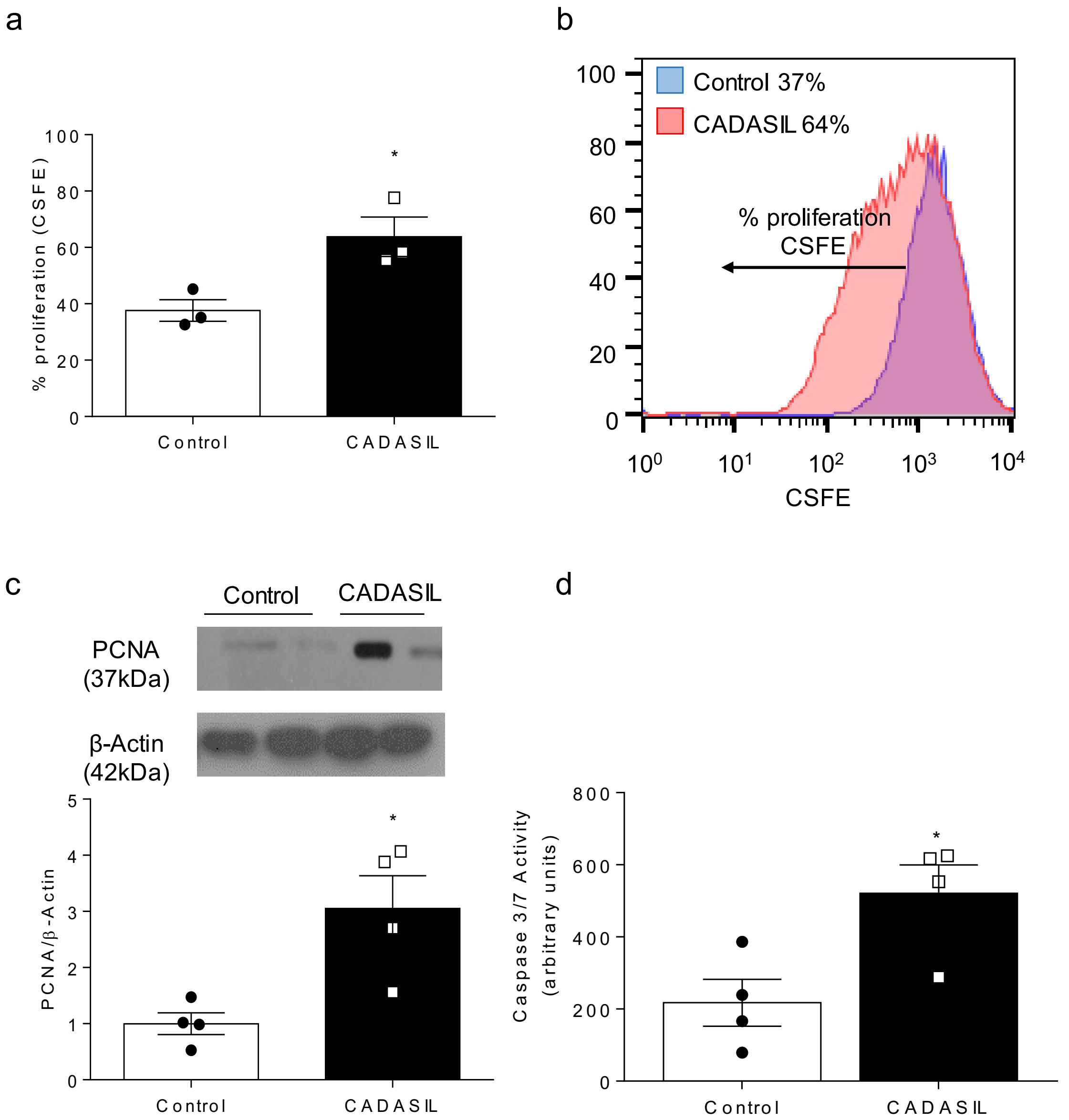

Figure S2. VSMC growth and apoptosis in CADASIL and control groups. (a, b) VSMC proliferation was assessed using the carboxyfluorescein succinimidyl ester (CSFE) assay (n=3; Student's *t* test) and (c) by proliferating cell nuclear antigen (PCNA) expression detected by western blot. Protein expression was normalised to β-actin (n=4; Student's *t* test). (d) An index of apoptosis was assessed by measuring caspase 3/7 activity assay in VSMCs from control and CADASIL subjects (n=4/group; Student's *t* test). Bars represent the mean±SEM. *p<0.05 vs Control.

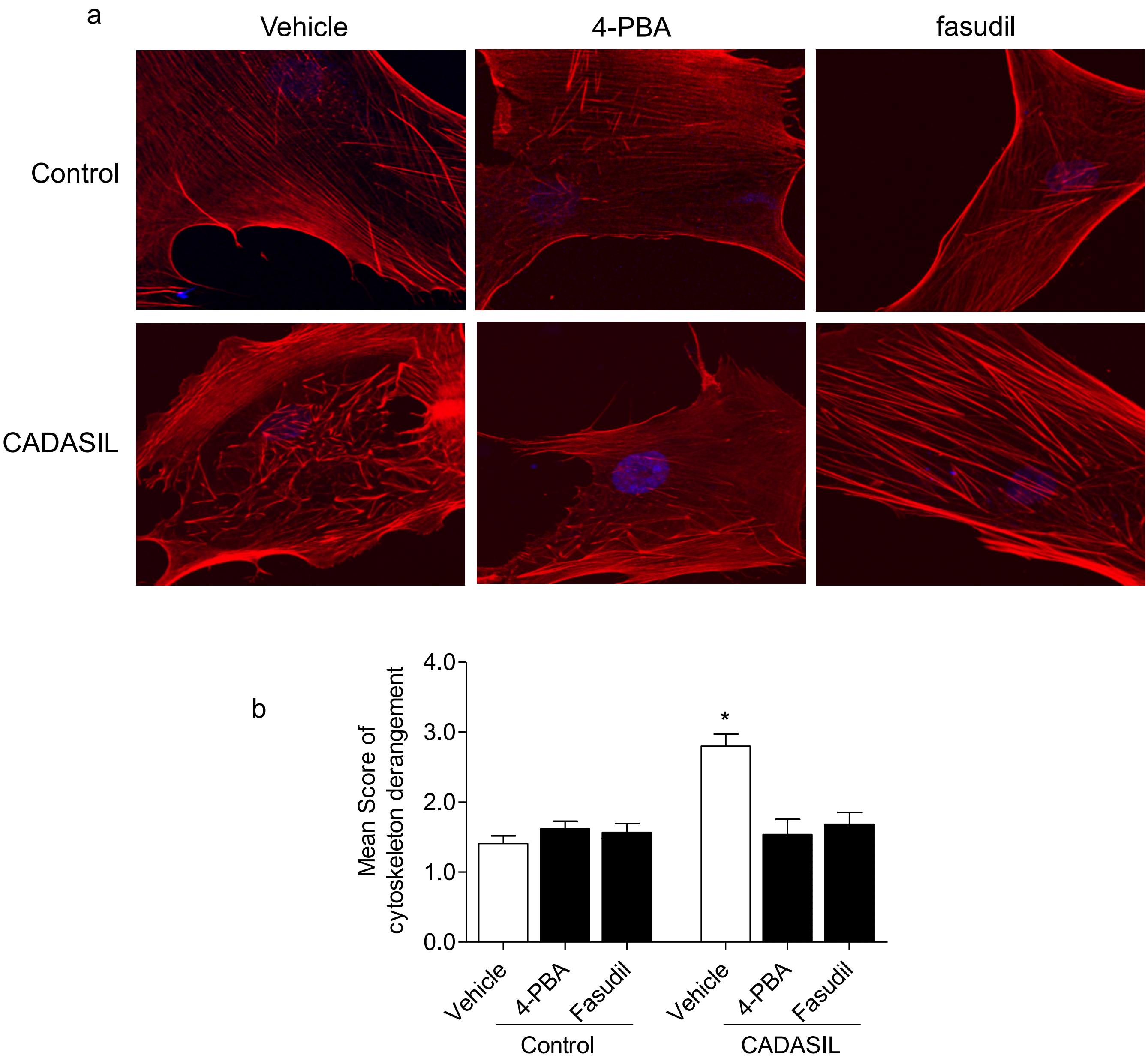

Figure S3. **Phalloidin staining of actin filaments in VSMCs from CADASIL and control subjects**. Cytoskeletal organisation was assessed in VSMCs by phalloidin staining of actin filaments (F-fibers). Nuclei were stained with DAPI. Immunofluorescence images of VSMCs treated with vehicle, 4-PBA or fasudil are presented in (a). Fluorescence imaging was performed using a Zeiss confocal system (LSM500). DAPI was excited at 405 nm and phalloidin at 535 nm. Semi-quantitative analysis was performed using a scoring system as detailed in the methods section. Relative fluorescence is expressed semiquantitatively and a relative score obtained, presented graphically in (b). Each experimental group was imaged in duplicate with a minimum of 40 images analysed.

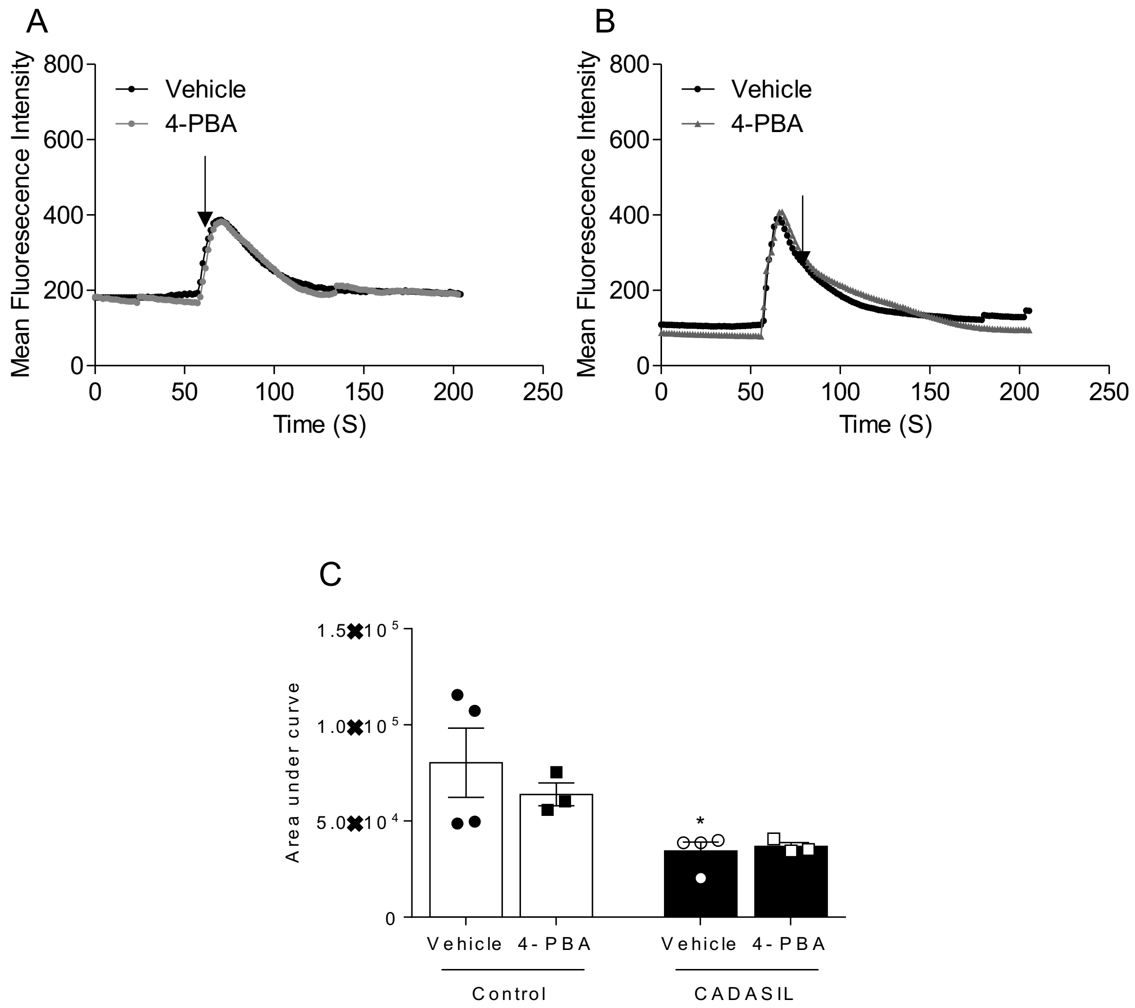

Figure S4. **ER stress inhibition has no effect on altered Ca^{2+} response to Ang II in CADASIL VSCMs.** Intracellular Ca^{2+} levels in response to Ang II (10^{-7} mol/L) were assessed using the Cal-520 fluorescent probe. Control and CADASIL VSMCs were pretreated with 4-PBA (10^{-3} mol/L). (a) Representative Ca^{2+} responses to Ang II in the absence and presence of 4-PBA in CADASIL VSMCs. (b) Represenattive Ca^{2+} responses to Ang II in the absence and presence of 4-PBA in control VSMCs. (c). Bar graphs are the mean±SEM of Ang II-induced Ca^{2+} responses presented as the area under the curve. Arrow indicates time of Ang II addition. Results are expressed as mean±SEM (n=3-4/group); One-way ANOVA with Bonferroni post-test. *p<0.05 vs Control counterpart.

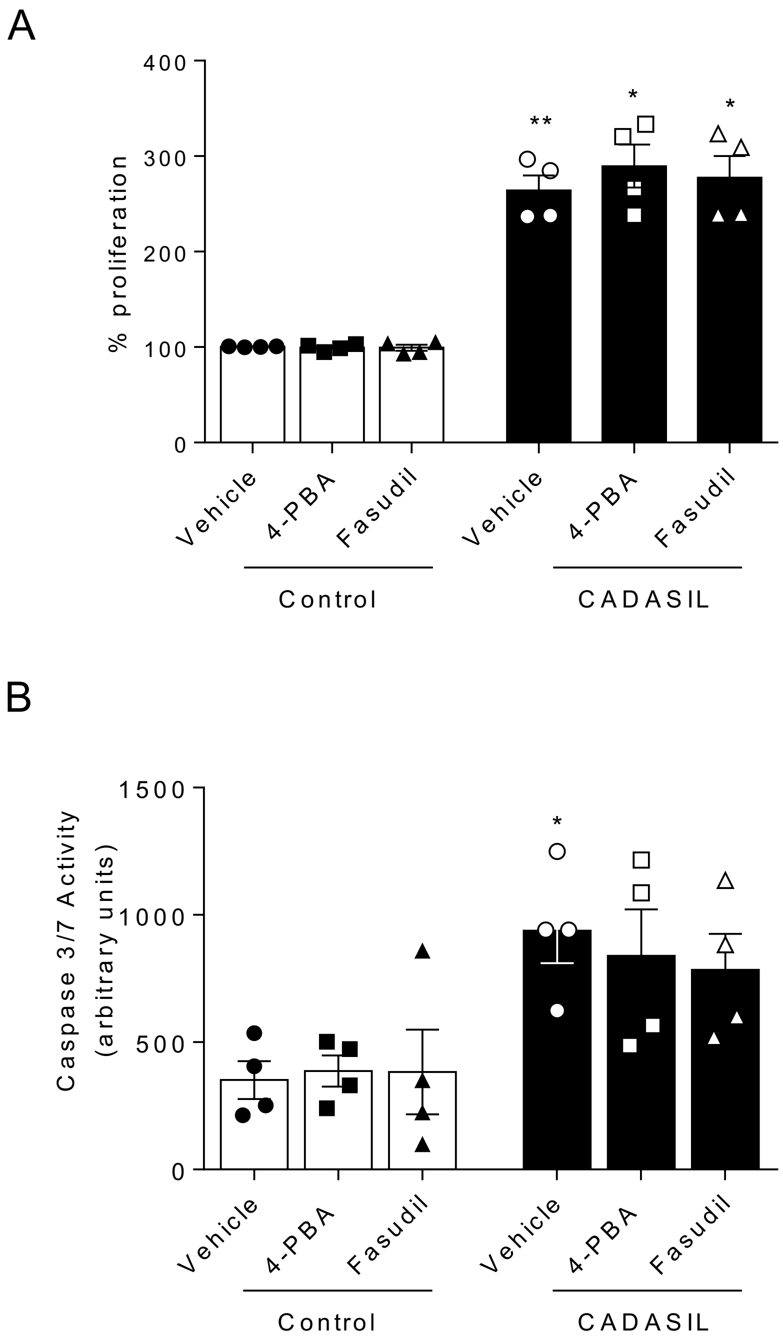

Figure S5. Inhibition of ER stress and Rho kinase has no effect on enhanced apoptosis and proliferation in CADASIL VSMCs. 4-PBA (10^{-3} mol/L) and fasudil (10^{-5} mol/L) pretreatment did not alter (a) enhanced CADASIL VSMC proliferation, as assessed by CSFE assay or (b) apoptosis, as assessed by Caspase 3/7 activity. Results are expressed as mean±SEM (n=4/group). One-way ANOVA with Bonferroni post-test). *p<0.005, **p<0.001 vs Control counterparts.
